# Inter-Homolog Crossing-Over and Synapsis in *Arabidopsis* Meiosis Are Dependent on the Chromosome Axis Protein AtASY3

**DOI:** 10.1371/journal.pgen.1002507

**Published:** 2012-02-02

**Authors:** Maheen Ferdous, James D. Higgins, Kim Osman, Christophe Lambing, Elisabeth Roitinger, Karl Mechtler, Susan J. Armstrong, Ruth Perry, Mónica Pradillo, Nieves Cuñado, F. Chris H. Franklin

**Affiliations:** 1 School of Biosciences, University of Birmingham, Birmingham, United Kingdom; 2 Institute of Molecular Pathology (IMP), Vienna, Austria; 3 Institute of Molecular Biotechnology (IMBA), Vienna, Austria; 4 Departamento de Génetica, Facultad de Biología, Universidad Complutense de Madrid, Madrid, Spain; Stowers Institute for Medical Research, United States of America

## Abstract

In this study we have analysed AtASY3, a coiled-coil domain protein that is required for normal meiosis in *Arabidopsis*. Analysis of an *Atasy3-1* mutant reveals that loss of the protein compromises chromosome axis formation and results in reduced numbers of meiotic crossovers (COs). Although the frequency of DNA double-strand breaks (DSBs) appears moderately reduced in *Atasy3-1*, the main recombination defect is a reduction in the formation of COs. Immunolocalization studies in wild-type meiocytes indicate that the HORMA protein AtASY1, which is related to Hop1 in budding yeast, forms hyper-abundant domains along the chromosomes that are spatially associated with DSBs and early recombination pathway proteins. Loss of AtASY3 disrupts the axial organization of AtASY1. Furthermore we show that the AtASY3 and AtASY1 homologs BoASY3 and BoASY1, from the closely related species *Brassica oleracea*, are co-immunoprecipitated from meiocyte extracts and that AtASY3 interacts with AtASY1 via residues in its predicted coiled-coil domain. Together our results suggest that AtASY3 is a functional homolog of Red1. Since studies in budding yeast indicate that Red1 and Hop1 play a key role in establishing a bias to favor inter-homolog recombination (IHR), we propose that AtASY3 and AtASY1 may have a similar role in *Arabidopsis*. Loss of AtASY3 also disrupts synaptonemal complex (SC) formation. In *Atasy3-1* the transverse filament protein AtZYP1 forms small patches rather than a continuous SC. The few AtMLH1 foci that remain in *Atasy3-1* are found in association with the AtZYP1 patches. This is sufficient to prevent the ectopic recombination observed in the absence of AtZYP1, thus emphasizing that in addition to its structural role the protein is important for CO formation.

## Author Summary

Homologous recombination (HR) during prophase I of meiosis leads to the formation of physical connections, known as chiasmata, between homologous chromosomes (homologs). Chiasmata are essential for accurate homolog segregation at the first meiotic division. HR is initiated by the formation of DNA double-strand breaks (DSBs). As DNA replication prior to meiosis results in the duplication of each homolog to form two identical sister chromatids, a DSB in one sister chromatid could potentially be repaired using the other as the repair template rather than one of the two non-sister chromatids of the homolog. If this route were predominant, the formation of chiasmata would be disfavored and chromosome segregation would be compromised. However, during meiosis there is a strong bias towards inter-homolog recombination (IHR). In this study we have identified AtASY3, a component of the proteinaceous axes that organize the chromosomes during meiosis in *Arabidopsis*. We find that AtASY3 interacts with AtASY1, a previously identified axis protein that is essential for crossover formation. We show that loss of AtASY3 disrupts the axis-organization of AtASY1. This results in a substantial reduction in chiasmata, and there is extensive chromosome mis-segregation. We propose that loss of AtASY3 affects the efficiency of the inter-homolog bias.

## Introduction

Meiotic recombination is initiated by the formation of Spo11-catalysed DSBs during early prophase I [1]. Each break is resected to produce 3′ single-stranded DNA tails which then interact with the RecA homologs Rad51 and Dmc1 to form nucleoprotein filaments. The filament on one side of the break then invades the homologous duplex DNA on either of the two non-sister chromatids resulting in strand displacement to form a displacement loop (D-loop). Extension of the invading strand via DNA synthesis increases the size of the D-loop, thus enabling the capture of the 3′-end of the DNA from the other side of the DSB. Further DNA synthesis and ligation of the DNA ends leads to the formation of two four-way junctions termed a double-Holliday junction (dHj) that, on resolution, results in the formation of a CO (reviewed in [2]).

An important feature of meiotic recombination is its close coordination with the alignment, pairing and synapsis of homologous chromosomes during prophase I (reviewed in [3]). Mutants that are defective in chromosome axis or SC morphogenesis exhibit a profound effect on recombination and subsequent CO formation. For example, during meiosis in budding yeast IHR predominates over inter-sister chromatid recombination, this bias being in part dependent on the chromosome axis proteins Hop1 and Red1 [4]. The Hop1-related proteins AtASY1 and Hormad1/2 are thought to perform the equivalent role in *Arabidopsis* and mouse respectively [5]–[7]. It is proposed that AtASY1 is essential for AtDMC1-dependent IHR. In the absence of AtASY1, the association of AtDMC1 with the early recombination intermediates appears compromised such that virtually all IHR is aborted [8]. Mutation of the budding yeast SC transverse filament gene *ZIP1* results in a failure of CO-designated intermediates to progress to form COs [9]. CO formation is also affected, albeit in a distinct manner, in the corresponding *Arabidopsis* and rice mutants [10], [11]. In the case of *Arabidopsis* lacking the Zip1 homolog, AtZYP1, there is a moderate reduction in CO frequency which is accompanied by the occurrence of recombination between non-homologous chromosomes. In rice, mutation of the *ZEP1* gene leads to an apparent increase in CO/chiasma frequency. The interrelationship between recombination proteins and meiotic chromosome organization is further emphasized in a study in the filamentous fungus *Sordaria macrospora* [12]. This revealed that, in addition to their previously described recombination functions, Mer3, Msh4 and Mlh1 have roles in ensuring accurate juxtaposition of the homologous chromosomes. Nevertheless, understanding the functional interrelationship between the recombination machinery and the chromosome axes and SC and the extent to which it is conserved between species has proved challenging. Although the structural organization of meiotic chromosomes is conserved, the chromosome axis and SC proteins exhibit a high level of primary sequence divergence. This limits the efficacy of straightforward homology searches as a route to identification of homologues in different species [13] and raises the question of how functionally related these proteins may be.

As one approach to overcome this problem we have begun to make an inventory of proteins present in meiocytes from *Arabidopsis* and the closely related species, *Brassica oleracea*, in order to analyze novel meiotic proteins and identify meiotic protein complexes ([14], KO, KM, ER and FCHF unpublished). Used in combination with homology searches this has enabled us to identify an *Arabidopsis* meiosis-specific coiled-coil protein, AtASY3. Analysis of AtASY3 has revealed that it is a component of the chromosome axes during meiotic prophase I. We demonstrate that loss of AtASY3 compromises AtASY1 localization leading to a reduced level of CO formation and a defect in chromosome synapsis. Our study provides further insights into the role of chromosome axis-associated proteins and the SC in the control of CO formation.

## Results

### Reduced fertility and meiotic defects in plants lacking the coiled-coil protein AtASY3

Analysis of proteins from *B. oleracea* meiocytes by mass spectrometry (MS) resulted in the detection of 4 peptides with homology to the predicted product of the *Arabidopsis* gene, At2g46980 (Figure S1A). We noted that At2g46980 (referred to hereafter as *AtASY3*) is predicted to encode an 88 kDa protein with a coiled-coil domain in its C-terminus region (Figure S1B). A database search revealed that the predicted coiled-coil protein was most closely related to a rice meiotic gene PAIR3 (25.6% identity) and had weak homology to Red1 from budding yeast (16.4% identity) [15]–[18]. RT-PCR analysis indicated that *AtASY3* was transcribed in reproductive tissues but not in vegetative tissues suggesting a potential role during the reproductive stage of development (Figure S1C). Thus we decided to characterize *AtASY3* further, to determine whether it encoded a meiotic protein and to establish its role.

We obtained three T-DNA insertion lines of *AtASY3* from the Nottingham Arabidopsis Stock Centre (NASC) (Figure S1B). Molecular characterization of each line confirmed the positions of the T-DNA insertions within *AtASY3* (Figure S2). Homozygous plants from each line showed normal vegetative growth but fertility was reduced by around 75% (Figure S3A).

**Figure 1:**
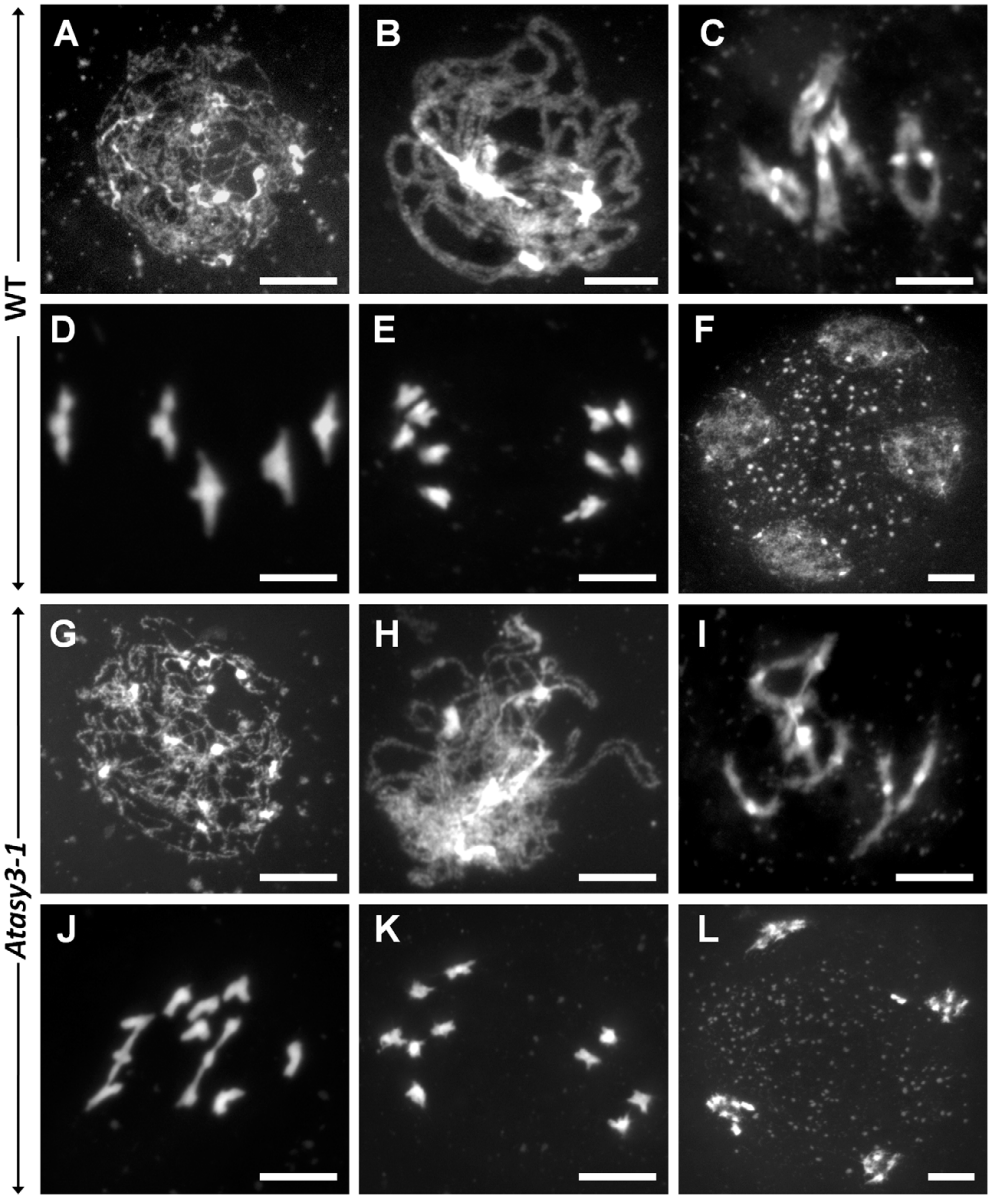
Meiotic stages. From wild-type (A–F) and *Atasy3-1* (G–L) pollen mother cells. (A,G) Leptotene. (B,H) Pachytene. (C,I) Diakinesis. (D,J) Metaphase I. (E,K) Dyad. (F,L) Tetrad. Early prophase I stages in *Atasy3-1* are similar to that of wild-type, however, at pachytene (H) homologous chromosomes fail to undergo full synapsis in *Atasy3-1*. Some univalents are present at diakinesis (I) and metaphase I (J) in *Atasy3-1* which can lead to chromosome mis-segregation at meiotic divisions and give rise to unbalanced dyad (K) and tetrad (L) nuclei. Bar, 10 µm.

The reduction in fertility was consistent with a defect in meiosis. To confirm this, DAPI-stained chromosome spread preparations from pollen mother cells (PMCs) were examined by fluorescence microscopy. Since the three lines were cytologically indistinguishable, the analysis of *Atasy3-1* is presented in Figure 1 and *Atasy3-2* and *Atasy3-3* are shown in Supplementary Figure S3B. Chromosome behavior was apparently normal from G2 through early prophase I (Figure 1A, 1G). However, normal pachytene nuclei were not observed (Figure 1B, 1H). As the chromosomes began to condense during late diplotene/diakinesis it became clear that a proportion of the homologous chromosome pairs had failed to form chiasmata (Figure 1C, 1I). This was confirmed by the presence of univalent chromosomes at metaphase I leading to mis-segregation at both meiotic divisions resulting in the formation of aneuploid tetrads (Figure 1D–1F, 1J–1L).

That the meiotic phenotype was due to mutation of *AtASY3* was confirmed by an allelism test in which a homozygous *Atasy3-1*/*Atasy3-2* double mutant was found to exhibit meiotic defects indistinguishable from the parental lines (Figure S3A–S3C) and a complementation test in which a full length *AtASY3* cDNA cloned under the control of the *AtDMC1* promoter in pPF408 [19], was found to restore normal meiosis in *Atasy3-1* (Figure S3D–S3I).

### Cytological analysis of AtASY3 and AtASY1 localization during meiotic prophase I in wild type

**Figure 2:**
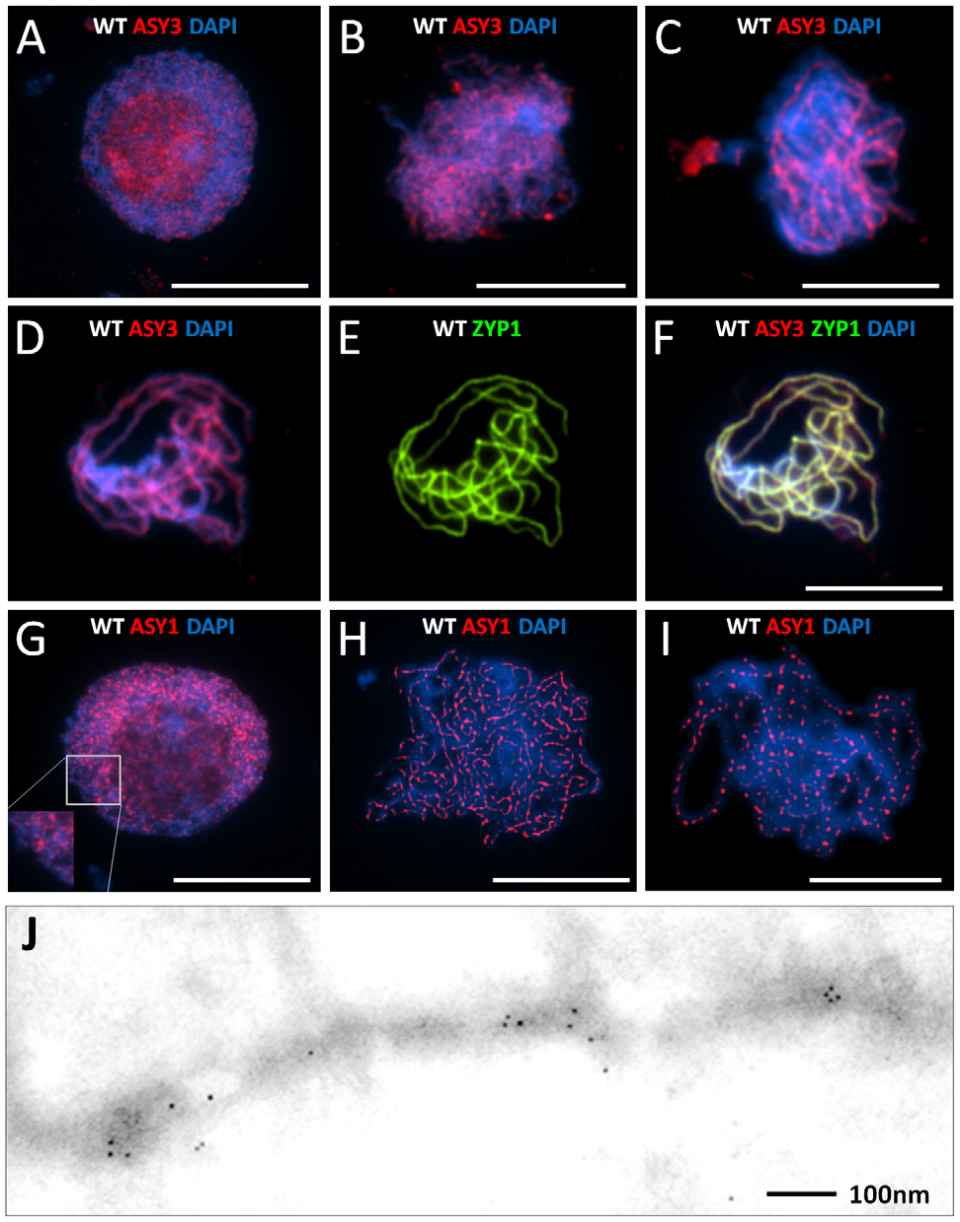
Immunolocalization of AtASY3 and AtASY1. Immunolocalization of AtASY3 (red) on DAPI stained (blue) wild-type meiocytes during meiotic prophase I (A–F). (A) Late G2, (B) Leptotene, (C) Zygotene, (D) Pachytene, dual localization with AtZYP1 (green) (E) and merged image (F). Immunolocalization of AtASY1 (red) on DAPI stained wild-type meiocytes during prophase I (G–I). (G) Late G2, (H) Leptotene (deconvoluted image), (I) Pachytene (deconvoluted image). Bar, 10 µm. (J) Immunogold labelling of AtASY1 on *Crepis capillaris* at meiotic prophase I. Bar 100 nm.

The distribution of AtASY3 was studied by immunolocalization on chromosome spread preparations of wild-type PMCs using anti-AtASY3 antibody (Figure 2). AtASY3 foci were first detected on the chromatin at late G2 together with accumulation of the protein in the nucleolus (Figure 2A). At leptotene the nucleolar signal of AtASY3 disappeared and the protein was detected along the chromosome axes (Figure 2B). This persisted through zygotene and pachytene during which partial colocalization with the SC transverse filament protein AtZYP1 [10] was observed (Figure 2C–2F).

In wild-type meiocytes at the transition from late G2 to leptotene, which is cytologically typified by a prominent centralized nucleolus, numerous chromatin-associated foci and short stretches of AtASY1 staining were observed (Figure 2G). By leptotene these developed into a linear, yet not entirely uniform, signal along the axes. Analysis using deconvolution software (see Materials and Methods) revealed that the variation in signal intensity at leptotene arises because AtASY1 is distributed along the axes as a series of diffuse hyper-abundant patches or domains separated by stretches of lower abundance (Figure 2H). This is accentuated at pachytene when the DAPI-stained chromosomes appear as thick, rope-like structures. At this stage the AtASY1 signal is depleted along the axis and the domains become foci-like in appearance (Figure 2I). The foci appear relatively evenly distributed and consistent in number (Mean number of foci per nucleus = 160; n = 10). The tendency for AtASY1 to form domains along the axis is supported by electron microscopy (EM) studies in the plant *Crepis capillaris*. Immunologold localization of ASY1 in chromosome spread preparations of *C. capillaris* meiocytes reveals that the gold particles form discrete axis-associated clusters rather than an evenly distributed signal (Figure 2J).

### Organization of the chromosome axis proteins and synaptonemal complex in *Atasy3-1*

**Figure 3:**
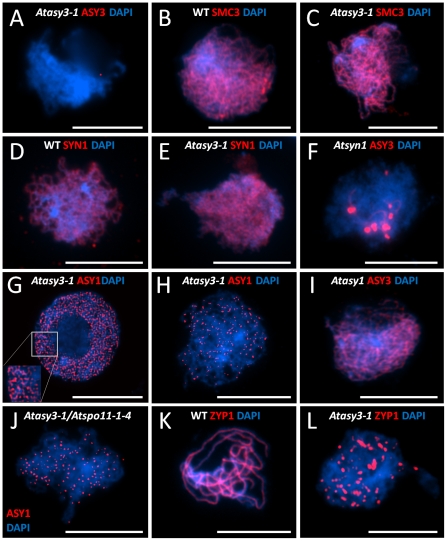
Immunolocalisation of chromosome axis proteins on *Arabidopsis* wild-type and chromosome axis mutants. At prophase I (A) AtASY3 (red) on *Atasy3-1*, (B) AtSMC3 (red) on wild-type and (C) *Atasy3-1*, (D) AtSYN1 (red) on wild-type and (E) *Atasy3-1*. (F) AtASY3 (red) on an *Atsyn1* mutant, G) AtASY1(red) on *Atasy3-1* at late G2 and (H) at late prophase I, (I) Immunolocalization of AtASY3 (red) on an *Atasy1* mutant at leptotene, (J) AtASY1(red) on an *Atspo11-1-4/Atasy3-1* mutant at late prophase I, (K) Immunolocalization of AtZYP1 (red) on wild-type at pachytene and (L) on *Atasy3-1* at late prophase I. DNA is stained with DAPI (blue) for all. Bar, 10 µm.

Application of anti-AtASY3 antibody to prophase I spread preparations of chromosomes from *Atasy3-1* PMCs did not result in any AtASY3 signal (Figure 3A). This confirmed the specificity of the anti-AtASY3 antibody and supported the RT-PCR analysis which indicated that the *AtASY3* transcript was absent in the mutant lines (Figure S1D).

Since AtASY3 localized to the chromosome axes during prophase I, we investigated the effect of loss of the protein in *Atasy3-1* on other axis components. Immunolocalization of the cohesin proteins AtSMC3 and AtSYN1, the *Arabidopsis* ortholog of the budding yeast meiotic cohesin Rec8 [20], [21], on spread preparations of *Atasy3-1* PMCs was indistinguishable from wild-type. Both proteins were detected as linear chromosome axis-associated signals during prophase I (Figure 3B–3E), suggesting global sister chromatid cohesion is present. In contrast, localization of AtASY3 was dependent on the cohesin complex, as it was completely disrupted in an *Atsyn1* mutant (Figure 3F).

Localization of AtASY1 in *Atasy3-1* meiocytes at late G2/early leptotene was similar to wild-type, with numerous chromatin-associated foci and short stretches of signal observed (Figure 3G). As prophase I progressed, AtASY1 co-localized with the axes. However, rather than forming a linear signal with the underlying domain organization observed in wild-type, the protein was detected as discrete, evenly-distributed foci which persisted until the chromosomes began to condense at the end of prophase I (Figure 3H). These appeared rather heterogeneous in shape and size. The mean number of the AtASY1 foci was 69 per nucleus (n = 30), but there was considerable variation between individual nuclei, with the number ranging from 39–115.

In contrast to the aberrant localization of AtASY1 in *Atasy3-1*, that of AtASY3 in an *Atasy1* mutant was indistinguishable from wild-type. This suggests that while normal localization of AtASY1 is dependent on AtASY3, this relationship is not reciprocal (Figure 3I). The same situation has been observed in budding yeast where Hop1 loading requires Red1 but not vice-versa [22].

Previously we have shown that the association of AtASY1 with the chromosome axes is independent of AtSPO11-induced DSB formation [8]. Consistent with this we observed that the axis-associated AtASY1 foci remained in an *Atasy3-1/Atspo11-1-4* double mutant (Figure 3J). As anticipated, the double mutant failed to form chiasmata, confirming that those detected in *Atasy3-1* are DSB-dependent (Figure S4B A,B).

As the initial cytological analysis of *Atasy3-1* indicated a defect in chromosome synapsis we investigated this in more detail. In wild-type meiocytes, the SC transverse filament protein, AtZYP1, polymerized to form the linear central region of the SC, such that at pachytene each pair of homologous chromosomes was fully synapsed (Figure 3K) [10]. In *Atasy3-1* the linearization of AtZYP1 to form a continuous SC did not occur. In most cases the AtZYP1 signals remained as foci or on occasion formed short stretches that were often abnormally thick and distorted in appearance. In some instances these structures could represent the accumulation of polycomplexes, nucleating at sites where AtZYP1 was unable to polymerize correctly along the lateral elements of the paired homologs (Figure 3L).

**Figure 4:**
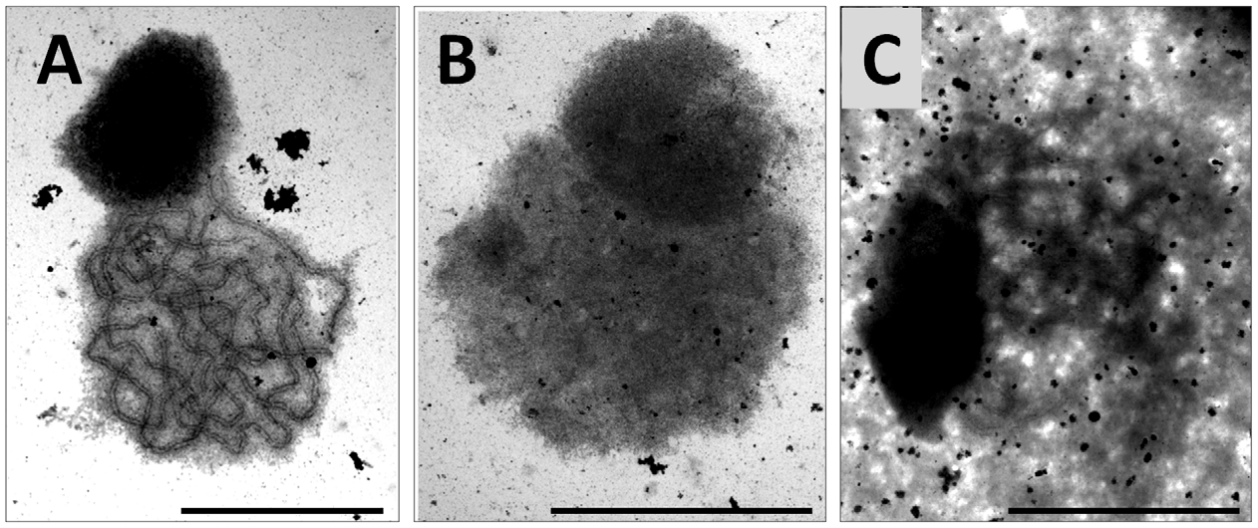
Electron micrographs of silver stained meiotic prophase I nuclei in wild-type and *Atasy3-1*. (A) wild-type, (B) *Atasy3-1* (C) *Atasy3-1* (modified method) showing aligned stretches of axis (arrow). Bar, 10 µm.

That SC formation was disrupted in *Atasy3-1* was supported by the analysis of silver-stained chromosome spread preparations using electron microscopy. In wild-type, fully synapsed homologous chromosomes were observed at pachytene (Figure 4A). In chromosome spread preparations of the *Atasy3* mutants using the same conditions the nuclei were diffuse and the chromosome axes could not be readily discerned (Figure 4B). However, by modifying the chromosome spreading conditions (see Materials and Methods) it was possible to detect nuclei where more extensive regions of axis were visible (Figure 4C). In some cases these were aligned, although the spacing often appeared variable. These observations support the immunolocalization studies that indicated that SC formation was disrupted. They also suggest that although chromosome axes are formed in *Atasy3-1*, there is likely some structural defect, possibly making them more susceptible to fragmentation by the chromosome spreading procedure. Alternatively, axis formation may be incomplete in the mutant.

### Chiasma frequency is significantly reduced in *Atasy3* mutants

To quantify the reduction in COs in the *Atasy3* mutants we analysed the chiasma frequency and distribution in 50 *Atasy3-1* PMCs. This revealed nuclei containing 0–6 chiasmata with an overall mean chiasma frequency of 3.3 (Figure 1J, Figure S4A, Table S1). Similar results were obtained for *Atasy3-2* (3.17 n = 50) and *Atasy3-3* (3.32 n = 50). These observations contrast with wild-type nuclei which contain 8–12 chiasmata with an overall mean of 9.76 [23]. Inspection of the chiasma distribution in the *Atasy3* mutants revealed that 74.8% of the residual chiasmata were localized to the distal regions of the chromosomes. This figure is unchanged from that of wild-type (73.8% n = 50) (Table S1).

To further analyze the functional relationship between AtASY3 and AtASY1 we constructed an *Atasy3-1/Atasy1* double mutant and compared the effect on chiasma formation to that in the *Atasy3-1* and *Atasy1* single mutants. This revealed a reduction in the mean chiasma frequency from 3.3 (n = 50) observed in *Atasy3-1* to 1.78 (n = 50; P<10^−7^) in *Atasy3-1/Atasy1*, but no significant difference between the double mutant and *Atasy1* (1.88 n = 50; P = 0.681) (Figure S4B C,D). Thus AtASY1 is epistatic to AtASY3 with regard to CO formation, whereas the relationship is reversed in terms of protein loading. A similar relationship exists for DSB formation and Red1 and Hop1 loading in budding yeast [22], [24]. Whereas Hop1 loading is greatly reduced in a *red1* mutant, but not vice-versa, a *hop1* mutant exhibits a stronger defect in DSB formation. Our data suggest that the higher CO frequency in *Atasy3-1* as compared to *Atasy3-1/Atasy1* may be attributable to the AtASY1 foci that remain in the single mutant, but that this is insufficient to promote wild-type levels of COs. However, this interpretation assumes that immunolocalization detects all the axis-associated AtASY1 that is present in *Atasy3-1*.

In budding yeast a group of proteins referred to as ZMM, comprising Zip1, Zip2, Zip3, Zip4, Msh4, Msh5 and Mer3, are crucial for the formation of interference-sensitive COs [9]. Homologs of the *ZMM* genes have been identified in *Arabidopsis* where their mutation results in a ∼85% reduction in CO formation [reviewed in 25]. The remaining COs (∼15%) exhibit a random numerical distribution based on a Poisson analysis [26], [27]. Since loss of AtASY3 resulted in a substantial reduction in chiasmata/COs we surmised that the protein was required for the formation of normal levels of *ZMM-*dependent COs. To investigate this, an *Atasy3-1/Atmsh4* double mutant was constructed and the chiasma frequency determined to establish if loss of AtASY3 resulted in any reduction in chiasmata over that observed in *Atmsh4*. In a survey of 30 metaphase I nuclei from the *Atasy3-1/Atmsh4* line no chiasmata were detected, whereas the mean chiasma frequency in the *Atmsh4* mutant was 1.1 (n = 30) (Figure S4B E,F). This indicates that AtASY3 has a role in the formation of all meiotic COs.

### Loss of AtASY3 affects DSB formation and localization of recombination pathway proteins

**Figure 5:**
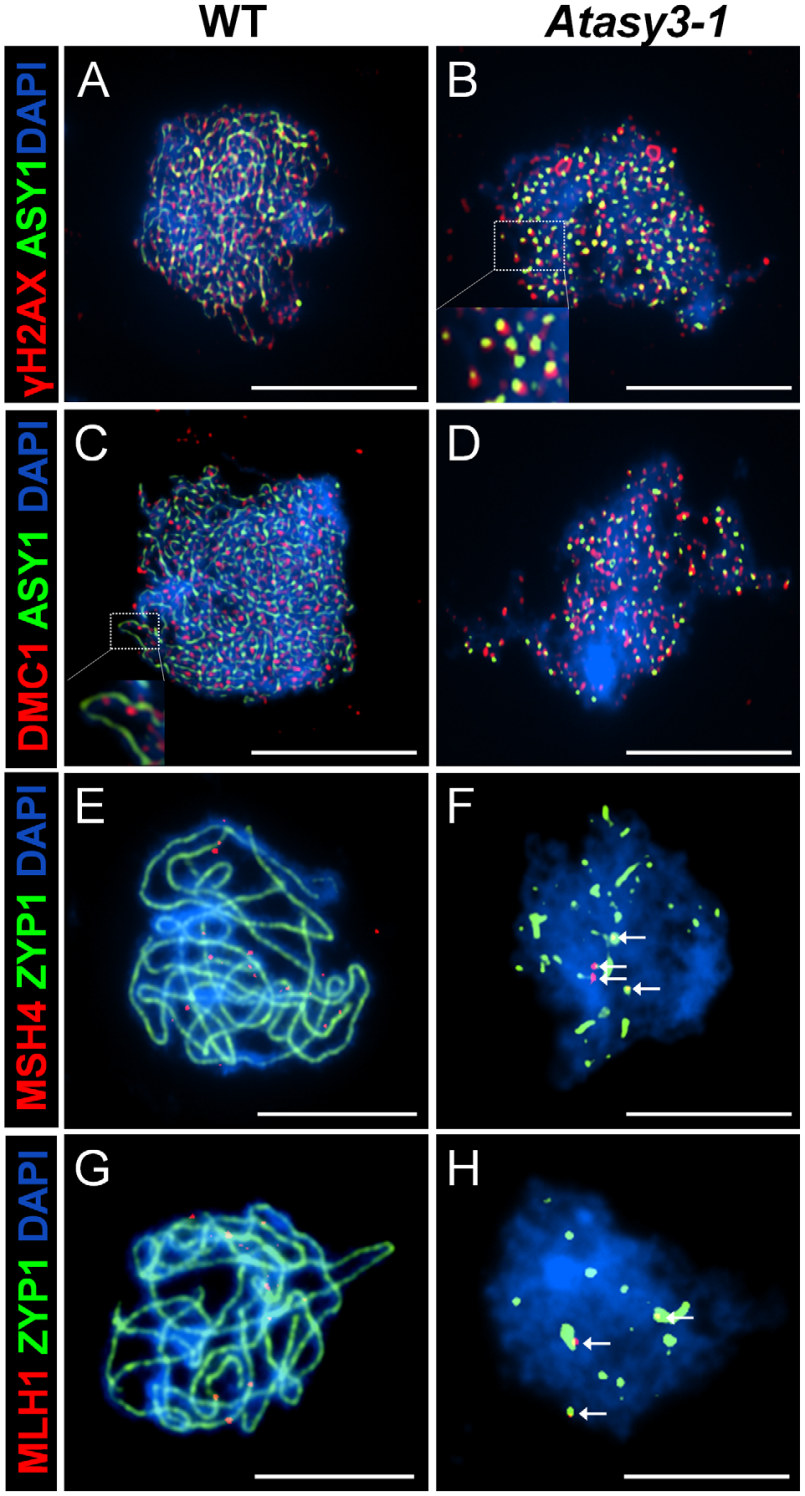
Dual immunolocalisation of AtASY1 and AtZYP1 with recombination pathway proteins in wild-type and *Atasy3-1* meiotic prophase I nuclei. (A) AtASY1 (green) and γH2AX (red) on wild-type and (B) *Atasy3-1* at leptotene, (C) AtASY1 (green) and AtDMC1 (red) on wild-type and (D) on *Atasy3-1* at leptotene. (E) AtZYP1 (green) and AtMSH4 (red) on wild-type and (F) on *Atasy3-1* (AtMSH4 arrowed). (G) AtZYP1 (green) and AtMLH1 (red) on wild-type and (H) on *Atasy3-1* (AtMLH1 arrowed). DAPI (blue) for all. Bar, 10 µm.

To address the basis for the reduced chiasma formation in *Atasy3-1*, we began by investigating the level of DSB formation in the mutant. The meiosis-specific histone H2AX is rapidly phosphorylated in chromatin surrounding the site of a DSB [28], [29], [30]. This phosphorylated form, γH2AX, can be detected by immunolocalization as foci in chromosome spread preparations from meiocytes. In wild-type nuclei a mean of 160.8 (n = 5) γH2AX foci were detected at leptotene (Figure 5A). In *Atasy3-1* the corresponding number was 114.2 (n = 5) (Figure 5B). This suggested that the formation of DSBs was significantly reduced in the *Atasy3-1* mutant (P<0.01). However, based on these observations we cannot exclude the possibility that the observed difference in the number of foci in the mutant compared to wild-type was due to an accelerated turn-over of DSBs. It also assumes that H2AX is phosphorylated at all DSBs in the mutant.

Immunolocalization of AtDMC1 and AtRAD51 [31], [32] revealed a significant reduction in the number of foci observed in *Atasy3-1* at leptotene relative to wild-type. In the case of AtDMC1, the figures for *Atasy3-1* and wild-type were 106.8 (n = 5) and 143.8 (n = 5) (P = 0.02) respectively (Figure 5C, 5D). For AtRAD51, the mean number of foci in *Atasy3-1* was 99.8 (n = 5) compared to 141 (n = 5) (P<0.01) in wild-type meiocytes (Figure S5A, S5B). Although the numbers of AtDMC1 and AtRAD51 foci only indirectly reflect the number of DSBs, these figures are consistent with the decrease in γH2AX foci observed in *Atasy3-1*.

The MutS homolog AtMSH4 is required for the formation of normal levels of interference sensitive COs [23]. In wild-type, AtMSH4 foci accumulate on the chromosomes at leptotene and these gradually reduce in number through zygotene. By early pachytene only a few remain and they continue to dissociate from the chromatin such that by late pachytene they have disappeared [23]. Similarly, in this study we observed a mean of 140 (n = 5) AtMSH4 foci in wild-type nuclei at leptotene which gradually reduced to around 10 foci detectable at early pachytene (Figure S5C; Figure 5E). A similar pattern of turnover of AtMSH4 foci was observed in *Atasy3-1*. However, the peak number of foci observed at each stage was lower than in wild-type. At leptotene a mean of 109.8 (n = 5) foci were recorded (Figure S5D). The foci reduced in number to between 0–4 at early pachytene and were found to associate with the stretches of AtZYP1 protein present in *Atasy3-1* (Figure 5F).

The MutL homologue, AtMLH1, is thought to mark the sites of COs/chiasmata [33]. Dual-immunolocalization of AtMLH1 and AtZYP1 on chromosome spread preparations revealed 8–12 AtMLH1 foci (mean = 9.8, n = 10) in wild-type nuclei at pachytene (Figure 5G). In *Atasy3-1* this was significantly reduced to a mean of 3.2 AtMLH1 foci (P<0.001; n = 10) per nucleus which is consistent with the observed chiasma frequency. These foci invariably co-localized with the patches of AtZYP1 that remained in the *Atasy3-1* meiocytes (Figure 5H).

These observations reveal a coordinate reduction in the number of γH2AX, AtDMC1, AtRAD51 and early AtMSH4 foci to around 60% of the number observed in wild-type. However, there must be an additional defect or defects since the overall number of crossovers, based on AtMLH1 foci and chiasma counts, is only around 30% of the wild-type level. Nevertheless, there is no overall repair defect as there is no evidence of chromosome fragmentation. Previously we have reported that loss of AtASY1 also leads to a major defect in CO formation, but with no obvious defect in DSB formation suggesting that DSBs occur at or near the wild-type level [8]. This was based on immunolocalization of γH2AX foci in squash preparations of meiocytes at early prophase I. Since our analysis of *Atasy3-1* was carried out using chromosome spread preparations we decided to determine the number of γH2AX foci in *Atasy1* using the same approach. This revealed that the mean number of γH2AX foci was 129.5 (n = 10) (Figure S5E, S5F). Thus as previously [8], there is no evidence of a major depletion of γH2AX foci in *Atasy1*, but a slight reduction cannot be excluded (P = 0.12).

### Interplay between the recombination and chromosome axis proteins

Dual localization of γH2AX and AtASY1 in wild-type nuclei revealed the γH2AX foci were adjacent to the hyper-abundant domains of AtASY1 and showed a slight tendency to overlap (Figure 5A). This observation is consistent with the proposal that the nascent DSB is tethered to the chromosome axis more or less coincident with its formation [4], [34], [35]. Similarly dual localization of AtDMC1 and AtASY1 revealed a close association of the AtDMC1 foci with the AtASY1-stained axis, but the signals were largely distinct and did not overlap (Figure 5C).

Despite the fact that AtASY1 was present as discrete foci rather than hyper-abundant domains in *Atasy3-1*, these were virtually all (97.4% n = 5) found in association with a γH2AX signal (Figure 5B). In a control designed to detect fortuitous co-localization (see Materials and Methods) this figure was 39% (n = 5). However, in the sample of cells analysed the mean number of γH2AX foci was greater than the number of AtASY1 foci (114.2 versus 87.8 respectively, n = 5). Hence, not all of the γH2AX foci co-localize with AtASY1 in *Atasy3-1*. The AtASY1 foci in *Atasy3-1* were also found in association with the AtDMC1 foci (95%, n = 5) (Figure 5D). However, in this case, unlike in wild-type, there was a tendency for signals to overlap. Hence it would appear that the two proteins end up localized at the same sites on the chromosomes but their spatial relationship may be perturbed in the absence of AtASY3.

We have previously shown that the stable association of AtDMC1 foci to the chromosome axes during early prophase I requires AtASY1 [8]. Since AtASY1 localization is disrupted in *Atasy3-1*, we investigated the localization of AtDMC1 in *Atasy3-1* in more detail. The chronology of AtDMC1 localization was determined by carrying out the immunolocalization of the protein together with prior BrdU pulse-labeling of the PMCs during meiotic S-phase as described previously [8]. This revealed that in *Atasy3-1*, *Atasy1* and wild-type maximum numbers of AtDMC1 foci accumulated on the chromosomes around 12 h following the BrdU-pulse. However, at 24 h while numerous AtDMC1 foci still remained in both *Atasy3-1* and wild-type, they were entirely absent in *Atasy1* (Figure S6). These data indicate that the rapid loss of AtDMC1 foci observed in *Atasy1* does not occur in *Atasy3-1*. That turnover of AtDMC1 foci in *Atasy1* is more rapid than in wild-type could suggest that some normal barrier to progression is absent, such that recombination proceeds but CO-designation is defective. Since the initial rate of accumulation of AtDMC1 is normal in *Atasy3-1* it would suggest that the reduced number of foci in the mutant may reflect a reduction in DSBs rather than an increase in turnover. Furthermore, it suggests that despite the overall depletion of AtASY1, the residual protein is sufficient to ensure that the temporal localization of AtDMC1 is similar to that in wild-type meiocytes.

### AtASY3 and AtASY1 and their Brassica homologs are able to interact

The finding that normal axis-association of AtASY1 was dependent on AtASY3 combined with the observation that the two proteins co-localized during prophase I suggested both a functional and possibly a direct physical inter-relationship between them. To obtain evidence that AtASY3 and AtASY1 may be components of a meiotic complex and possibly interact *in planta* we conducted a co-immunoprecipitation (CoIP) experiment using anti-AtASY1 antibody. Since extracting protein in sufficient quantities from *Arabidopsis* meiocytes is impractical due to the small size of the anthers, we used meiocyte extracts from *B. oleracea*. In previous studies we have shown that an anti-AtASY1 antibody recognizes the corresponding *Brassica* protein, BoASY1, which shares 83% amino acid sequence identity with AtASY1 [36]. Similarly, analysis of *BoASY3* from *B. oleracea* indicated that it encodes a protein with 77% sequence identity with its *Arabidopsis* counterpart (Figure S7) and that it localized to the chromosome axes during prophase I of meiosis (Figure S8). Co-immunoprecipitating proteins were identified by MS of tryptic peptides followed by searches against a combined *Brassica* database containing the BoASY1 and BoASY3 full-length sequences (see Materials and Methods for details). BoASY1 was identified as the top hit with 40 unique peptides corresponding to 397/599 amino acids (65% coverage) and BoASY3 as the second hit with 28 peptides corresponding to 297/776 amino acids (38% coverage) (Figure S9A). Both proteins were absent from the control sample. Thus it would seem probable that BoASY1 and BoASY3 are components of a complex and given the close conservation with *Arabidopsis*, is likely the case for AtASY1 and AtASY3.

**Figure 6:**
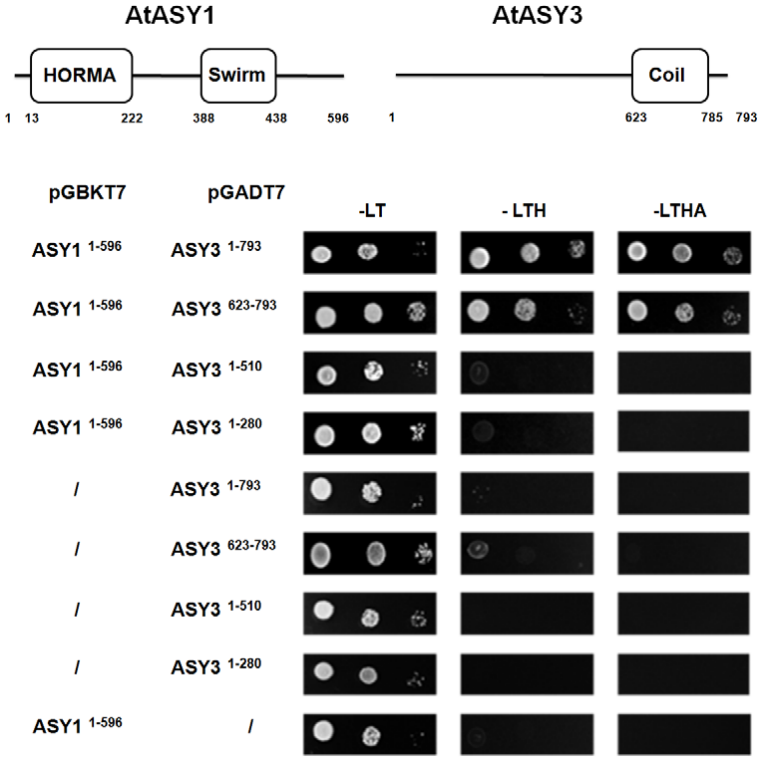
Yeast two-hybrid analysis of AtASY3 and AtASY1 with schematic illustration of predicted domains and relative positions of amino acid residues. Plasmid constructs were co-transformed into Y2HGold yeast cells before plating on SD -Leu/-Trp (-LT), SD -Leu/-Trp/-His (-LTH) and SD -Leu/-Trp/-His/-Ade (-LTHA). Growth on –LTH and –LTHA confirms a protein-protein interaction between AtASY1 and AtASY3 that is dependent on AtASY3 residues 623–793.

To determine if the proteins can directly interact, the full-length cDNAs of each gene were cloned as in-frame fusions with the GAL4 DNA binding domain (DBD) and the GAL4 activator domain (AD) in the yeast two hybrid plasmids pGBKT7 and pGADT7 respectively. Plasmids encoding the AtASY1-DBD and AtASY3-AD fusions and the reciprocal pair of constructs were co-transformed into yeast. At the same time control transformations, where one of the constructs was replaced by the corresponding empty vector, were also carried out. All plasmid combinations enabled the transformed yeast to grow on synthetic dropout medium (-Leu/-Trp) that selected the auxotrophic markers on the cloning vectors. When the plasmid combinations were tested on low stringency selective plates (-Leu/-Trp/-His) the yeast cells containing AtASY1 and AtASY3 as reciprocal DBD/AD fusions enabled significant growth at all dilutions. Some slight growth was also detected in controls containing the AtASY1-DBD and AtASY3-AD plasmids. However, under higher stringency selection (-Leu/-Trp/-His/-Ade) growth was entirely restricted to the combinations carrying both genes (Figure 6). Further experiments with a series of plasmids in which truncated regions of AtASY3 were fused to the GAL4 activator domain indicated that the interaction of AtASY3 with AtASY1 was dependent on amino acid residues 623–793 of AtASY3 which correspond to the predicted coiled-coil region (Figure 6). These data suggested that AtASY1 and AtASY3 can interact and likely do so *in planta*.

## Discussion

### AtASY3 is a component of the meiotic chromosome axes in *Arabidopsis*

We have identified AtASY3, a chromosome axis protein that is required for normal levels of COs and SC formation in *Arabidopsis*. AtASY3 is predicted to contain a coiled-coil domain towards the C-terminus. This structural feature is found in other meiotic proteins, such as Red1 in budding yeast, SCP3/Cor1 in mammals and OsPAIR3 in rice, that are components of the axial regions of the SC, yet on the basis of amino acid sequence homology are reported to have no close homologs in other species [15], [16], [18], [37], [38]. AtASY3 appears similar in this respect. Although it shares 77% sequence identity with BoASY3 from the closely related *B. oleracea*, the level of sequence identity with Red1 and OsPAIR3 is limited, at 16.4% and 25.6% respectively. This sequence divergence appears to be a recurrent feature of proteins that are components of the chromosome axes.

### DSBs appear reduced and crossover formation is compromised in *Atasy3-1*

In *Arabidopsis* the level of DSBs may be inferred from the number of γH2AX and/or AtDMC1 or AtRAD51 foci detected in meiocytes at early prophase I [8]. Our data suggested a consistent reduction in DSBs from 150–160 in wild-type meiocytes to about 100 in *Atasy3-1*, an overall reduction of around 33%. However, as DSB detection is indirect, we cannot formally rule out the possibility that not all DSBs are detected in the mutant. Nevertheless, as the number of γH2AX foci is very similar to those of the strand-exchange proteins this seems a reasonable conclusion.

The physical association of the recombination machinery and the chromosome axes has been known for many years [3], [34], [39]. Recent studies in budding yeast indicate that the DSB machinery becomes tethered to the chromosome axes prior to break formation and that the axis components Red1 and Hop1 are required for this [4], [35]. Analysis of *red1* mutants has revealed a defect in DSB formation [40]–[42]. It is conceivable that AtASY3 may play a similar role in axis organization that is crucial to enable normal levels of DSB formation. The reduction in foci corresponding to γH2AX and the strand-exchange proteins is consistent with a scenario whereby DSB formation occurs in the context of the axis, rather than the recombination complex associating with the axis following break formation, as in the latter instance, a reduction in γH2AX foci would not be anticipated. However, it remains possible that depletion of AtASY3 may induce a change in chromatin structure that in turn results in a reduction in DSBs.

Although DSB formation appears to be significantly reduced in *Atasy3-1*, any reduction due to the loss of AtASY1 appears more marginal. This is in broad agreement with our earlier study [8]. It is worth noting that this difference mirrors chromosome axis formation in the two mutants. Whereas loss of AtASY3 disrupts the axes, the *Atasy1* mutant has clearly defined axes, albeit with some minor discontinuities, seemingly indicating the importance of the chromosome axes for efficient DSB formation [43]. However, it contrasts with observations in budding yeast and mouse, where mutants lacking Hop1 and HORMAD respectively exhibit strongly reduced numbers of DSBs [44], [45]. At present the basis and significance of this difference remains unknown. It may reflect an underlying difference in the control of DSB formation, although the formation of DSBs in *hop1* and *red1* mutants in different budding yeast strains also shows some variation [40], [46].

A reduction in the mean chiasma/CO frequency to around 30% of the wild-type level was observed in all *Atasy3* lines. Analysis of the *Atmsh4/Atasy3-1* double mutant indicates that loss of AtASY3 compromises the formation of COs which are subject to CO interference, and also non-interfering COs. This would suggest that AtASY3 plays a crucial role at an early stage in the recombination pathway. A reduction in CO formation is also a characteristic of mutants in the axis-associated proteins Red1 and OsPAIR3 [18], [24], [40], [41]. A recent study in budding yeast has indicated that loss of Red1 results in a conversion from inter-homolog bias to inter-sister bias for DSB repair. It is proposed that this is due to a loss of constraint on Rec8 loading at the recombination site, thus favoring inter-sister recombination [4]. It is conceivable that a similar situation arises through the loss of AtASY3. If so, this could explain why there is a proportionally greater reduction in CO formation in *Atasy3-1* than might be expected from the apparent reduction in DSBs. Simple extrapolation would suggest that if the ratio of COs to non-COs was maintained in the mutant, the mean CO/chiasma frequency would be 6–7 rather than the 3.5 observed. This implies that there is a loss of inter-homolog bias and that a greater proportion of the DSBs are repaired via another route such as inter sister-chromatid exchange. Alternatively, loss of AtASY3 may result in preferential processing of some or all of the recombination intermediates to favor non-COs rather than COs. At present the data does not enable us to distinguish between these possibilities.

The observation that 75.7% of the chiasmata that remain in *Atasy3-1* are sub-telomeric/distal is consistent with previous studies in *Arabidopsis* showing that the telomeres cluster on the nucleolus during early prophase I and as a result the sub-telomeric regions of the homologous chromosomes are placed in proximity [47]. A similar situation was observed in *Atasy1* where virtually all the residual chiasmata are distal [48].

### Axis-association of AtASY1 is AtASY3-dependent and occurs in the absence of DSB formation

Deconvolution of the linear AtASY1 signal in wild-type meiocytes at leptotene through early pachytene revealed that it comprises evenly-spaced axis-associated domains of hyper-abundance interspersed with more lightly staining regions. This organization was supported by immunogold studies in meiocytes from *C. capillaris*. The number of AtASY1 domains appeared quite consistent, at around 160 per nucleus. The organization of AtASY1 is highly reminiscent of that of Hop1 which has been observed to form domains of alternating hyper-abundance and lower-abundance in budding yeast at early pachytene [49], [50]. Loss of AtASY3 resulted in a significant effect on the distribution of AtASY1, such that axis-associated foci were observed during prophase I. These were fewer in number than the AtASY1 domains observed in wild-type and this varied from cell to cell ranging from 39–115 in the sample examined. Nevertheless, dual-localization studies with γH2AX and AtDMC1 indicated that these AtASY1 foci coincided with sites of recombination. However, further investigation will be required to determine whether or not localization of AtASY1 at the axial region still occurs with normal spatial specificity in *Atasy3-1*. Studies in budding yeast initially reported that localization of the AtASY1 homolog, Hop1, was dependent on the chromosome axis protein Red1 [22]. However, more recently it was suggested that while normal levels of Hop1 loading require Red1, some Hop1 is loaded at the sites of DSBs independently [46]. Our observations suggest a similar relationship between AtASY1 and AtASY3.

In wild-type, the AtASY1-abundant regions along the axes at prophase I correlated both spatially and numerically with the γH2AX and AtDMC1 foci, suggesting that the DSBs are positioned within AtASY1-enriched domains. This is consistent with the proposed role of AtASY1 in promoting IHR [8]. Interestingly, whereas the γH2AX and AtASY1 signals overlapped, the AtDMC1 signal was adjacent to, but did not merge with the AtASY1 signal. Further study will be required to explore the significance of this observation. It appears that a similar spatial relationship between DSBs and AtASY1 is maintained in *Atasy3-1* as virtually all the AtASY1 foci in the mutant co-localize with γH2AX. Thus, it suggests that recruitment of AtASY1 and the recombination machinery to the axial region is spatially coordinated. However, these may not be interdependent events since localization of AtASY1 in the *Atspo11-1-4* null mutant appears, based on immunofluorescence, normal [8]. Similarly in this study, examination of AtASY1 localization in an *Atasy3-1/Atspo11-1-4* double mutant suggests that the AtASY1 foci observed in *Atasy3-1* are still formed and associated with the axial region. Nevertheless, further studies will be required to establish if the AtASY1 domains observed in *Atspo11-1-4* and *Atasy3-1/Atspo11-1-4* are identical to those in the wild-type. If so, then it suggests that AtASY1 is initially recruited to predetermined chromosomal regions that also encompass DSB hotspots, possibly establishing a spatial relationship favoring IHR. Alternatively, it is conceivable that the AtASY1 domain formation observed in wild-type may be guided or influenced by DSB formation or the pre-DNA break recombinosome complex. Studies in budding yeast have also led to the proposal that the interhomolog bias is established before DSB formation with enforcement of the bias occurring at the transition from the nascent DSB to a joint molecule recombination intermediate [41].

Although the majority of the AtASY1 foci in *Atasy3-1* were associated with γH2AX foci at early prophase I, there were additional γH2AX signals that did not colocalize. If loss of DSBs and the destabilization of AtASY1 localization observed in *Atasy3-1* occurs stochastically, then, given that formation of AtASY1 foci and DSBs does not appear to be co-dependent, one would expect to see a similar proportion of γH2AX and AtASY1 foci that were not associated with one another. As this does not appear to be the case, it seems possible that a sub-set of DSBs occur in regions that are not associated with AtASY1. It may simply be that although the AtASY1 foci that persist in *Atasy3-1* correspond to the position of the domains observed in wild-type they are substantially smaller. Hence a proportion of the γH2AX foci no longer co-localize despite the normal spatial recruitment of the recombination complexes to the chromatin. Alternatively, some DSBs may occur in regions of lower AtASY1 abundance. Previously, it has been proposed in budding yeast that some DSBs are formed at random sites that are not associated with DSB hotspots [41]. Hence, if the AtASY1 domains are coincident with hotspots, which would seem logical, this may also be the case in *Arabidopsis*.

### Evidence suggests that AtASY3 and Red1 share functional similarities

Despite amino acid sequence variation it has been suggested that Red1 and SCP3/Cor1 may be structural analogs [15]. Nevertheless, to date a functional ortholog of Red1 in a multi-cellular organism has not been reported. However, the studies described here suggest that AtASY3 has at least some functional similarity to Red1. The most compelling evidence arises from the close functional interrelationships that both proteins share with their corresponding HORMA domain proteins. Loss of Red1 and AtASY3 proteins results in a disruption of Hop1 and AtASY1 localization respectively, during prophase I [22]. In budding yeast Red1 has been shown to interact with Hop1 in co-immunoprecipitation experiments [46]. Yeast two-hybrid studies have shown that the 290 amino acids at the C-terminus of Red1 are essential for the interaction with Hop1. This part of the protein is predicted to form a coiled-coil domain. In this study MS analysis of proteins that were co-immunoprecipitated from *Brassica* meiocytes using an anti-AtASY1 antibody revealed a likely interaction between BoASY1 and BoASY3, the homologs of AtASY1 and AtASY3 respectively. That AtASY3 and AtASY1 can directly interact was confirmed by yeast two-hybrid analysis. Moreover further study revealed that the C-terminal of AtASY3 that contains a predicted coiled-coil region is essential for the interaction between the two proteins. Studies indicate that Red1 is required for normal levels of DSB formation. This also appears to be the case for AtASY3, although this effect does not appear as pronounced in *Atasy3-1* as that in a *red1* mutant where a reduction in DSB formation to ∼25% of wild-type has been reported [42].

It seems likely that PAIR3 in rice may also be a functional homolog of Red1. While a direct interaction with the rice HORMA domain protein OsPAIR2 has not yet been demonstrated, localization of OsPAIR2 is OsPAIR3 dependent and an *Ospair3* mutant has a similar phenotype to that of *Atasy3-1* [17], [18].

### SC nucleation is sufficient to prevent aberrant ectopic recombination

In addition to its structural role within the SC, the budding yeast protein Zip1 has been shown to play a key role in meiotic recombination [51], [52]. Zip1 together with other members of the ZMM group of proteins, Zip2, Zip3, Zip4, Msh4, Msh5 and Mer3, is crucial for the formation of interference-dependent COs [51]. In *Arabidopsis* loss of AtZYP1 only results in a modest reduction in chiasma frequency to about 80% of the wild-type level. However, many of the remaining chiasmata occur between ectopic chromosome regions, possibly between duplicated sequences that amount to around 60% of the *Arabidopsis* genome [10]. The studies described here revealed that loss of AtASY3 had a profound effect on the formation of the SC. In most nuclei it appeared that alignment of the chromosome axes was extensively disrupted and immunolocalization studies with the transverse filament protein AtZYP1 indicated little evidence of normal SC assembly. In general, nuclei contained a mixture of AtZYP1 foci or occasional short stretches which appeared abnormally thickened and deformed. Although *Atasy3-1* is essentially asynaptic, there is no evidence of the non-allelic recombination observed in plants lacking AtZYP1. The bivalents that remained in the *Atasy3-1* meiocytes at metaphase I comprised homologous chromosomes and there was no evidence of multivalent formation. Immunolocalization studies revealed a reduction in AtMLH1 foci that reflected the reduction in chiasmata in *Atasy3-1*. These AtMLH1 foci, which are thought to localize to CO sites [33], were invariably associated with the residual AtZYP1 present in the mutant. Hence, it would appear that the presence of AtZYP1 at the site of recombination is important for the prevention of non-allelic recombination, but extensive SC polymerization is not required. This finding provides further evidence that in addition to SC formation, AtZYP1 plays an important role in the formation of COs in *Arabidopsis*.

In summary, these studies provide further insight into meiotic CO formation. Moreover they emphasize that despite the lack of sequence homology between the chromosome axes components from different species, it seems likely that a close functional relationship remains.

## Materials and Methods

### Plant material and nucleic acid extraction

*A. thaliana* ecotype Columbia (0) was used for wild-type analysis. T-DNA insertion lines SALK_143676, SALK_050971 and SAIL_423_H01 were obtained from NASC for mutant analysis [53]. Plants were grown, material harvested and nucleic acid extractions were performed as previously described by Higgins *et al.* [23].

### T-DNA insertion site mapping

The T-DNA insertion site of the mutant lines was confirmed as previously described [23] (Figure S2). Details of the primers used are presented in Table S2.

### Complementation of Atasy3-1

Primers ASY3-CM-F1 and ASY3-CM-R1 (Table S2) were used to amplify the entire AtASY3 coding sequence with flanking 5′ and 3′ UTR regions from cDNA clone pda 19140 (Riken, Japan). The PCR product was cloned into the binary vector pPF408 [19] using SpeI sites incorporated into the primers. The construct was confirmed by sequencing. The binary plasmid construct was introduced into *Agrobacterium tumefaciens* LBA 4404 and plants transformed as previously described [23].

### RNA extraction and RT–PCR

RNA extraction and RT-PCR was carried out as previously described [54]. Details of the primers are given in Table S2.

### Nucleic acid sequencing

Nucleotide sequencing was carried out by the Genomics and Proteomics Unit, School of Biosciences, University of Birmingham, UK.

### Antibody production

Primers ASY3-AB-F1 and ASY3-AB-R1 (Table S2) were used to amplify a 702bp fragment comprising amino acid residues 560 to 793 of *AtASY3* from cDNA clone pda 19140 (Riken, Japan). The PCR product was cloned into the expression vector pET21b (Novagen) using *NdeI* and *Xhol* sites incorporated into ASY3-AB-F1 and ASY3-AB-R1 respectively. Recombinant His-tagged protein was isolated from *E. coli* BL21 (Novagen) under native conditions using Ni-agarose following the manufacturer's protocol (QIAGEN). Polyclonal antiserum against the recombinant protein was raised in rabbit (BioGenes GmbH, Germany).

### Cytological procedures

Cytological studies were carried out as previously described [23]. The following antibodies were used: anti-AtASY3 (rabbit, 1/200 dilution) anti-AtASY1 (rabbit/rat, 1/1000 dilution), anti-AtMSH4 (rabbit, 1/500 dilution), anti-AtZYP1 (rabbit/rat, 1/500 dilution), anti-AtSMC3 (rabbit 1/500 dilution), anti-AtSYN1 (rabbit 1/500 dilution), anti-AtDMC1 (rabbit 1/500 dilution), anti-AtRAD51 (rabbit, 1/500 dilution), anti-AtMLH1 (rabbit/rat, 1/200 dilution) and anti-γH2AX (ser 139, catalog no. 07-164 Upstate Biotechnology; rabbit, 1/100 dilution) [10], [23], [55]. Microscopy was carried out using a Nikon 90i Fluorescence Microscope (Tokyo, Japan). Image capture, image analysis and processing were conducted using NIS-Elements-F software (Nikon, Tokyo, Japan). Image deconvolution was carried out using the function “Mexican hat”. This allows better discrimination of the signals. This function performs filtration on the intensity component (or on every selected component - when working with multichannel images) of an image using convolution with 5×5 kernel. Mexican Hat kernel is defined as a combination of Laplacian kernel and Gaussian kernel it marks edges and also reduces noise. In double-staining experiments the level of chance overlap of foci was assessed using the misorientation method whereby one of the two images is rotated through180 degrees following which colocalizing foci are counted as previously described [56].

Electron microscopy and immunogold labeling was performed as previously described [36], [57] except for the modified *Atasy3* chromosome spread protocol where the detergent was Triton X-100 0.1%+Lipsol 0.05% and the digestion time was increased to 7 min.

Chiasma counts were carried out as previously described [58]. Chromosome spread preparations from PMCs at metaphase I were examined by light microscopy after fluorescence *in situ* hybridization (FISH) using 45S and 5S rDNA probes. The use of FISH enabled the identification of individual chromosomes. The overall shape of individual bivalents allowed the number and position of individual chiasmata to be determined and this was also informed by the position of the FISH signals.

### Statistical procedures

The statistical procedures were carried out as described previously [23].

### Proteomic analysis of Brassica meiocytes

AtASY3 peptides were initially identified in meiocyte extracts prepared from *Brassica oleracaea var. alboglabra* A12DHd as previously described [14]. Co-IP experiments were conducted on meiocytes extracted from the same material. Protein extraction was under non-denaturing conditions at 4°C. Briefly, tissue was powdered by grinding in liquid nitrogen, resuspended in IP Buffer (20 mM Tris-HCl pH 7.5, 150 mM NaCl, 10% glycerol, 2 mM EDTA, 0.1% NP40, protease inhibitor cocktail (Roche, #11836170001), phosphatase inhibitor (Thermo Scientific #78420)) and cell debris removed by centrifugation. Protein extracts were used immediately. Antibodies were cross-linked to Affi-Prep Protein A beads (Bio-Rad, #156-0006) using DMP (Sigma #D8388) and pre-eluted with glycine to remove any non-cross-linked antibody. Meiocyte extracts were pre-cleared by incubation with non-specific IgG. Parallel Co-IPs were carried out using affinity purified anti-AtASY1 antibody and an unrelated antibody as a control. Following washing to remove non-specific proteins, bound proteins were glycine-eluted, trypsin-digested and analysed on an LTQ-Orbitrap Velos mass spectrometer (Thermo Fisher Scientific). Since a complete sequence of the *Brassica oleracea* genome is currently unavailable, protein identification was carried out against a combined *Brassica rapa/napus/oleracea* database, downloaded from NCBI (http://www.ncbi.nlm.nih.gov) in 2010, into which the BoASY1 and BoASY3 full-length sequences had been manually inserted. For details of the MS analysis see Figure S9B.

### Yeast 2-hybrid analysis

Yeast two-hybrid screens were performed according to the Yeastmaker Yeast transformation System 2 manual (Clontech, USA). Briefly, Y2HGold yeast cells were co-transformed with pGADT7 and pGBKT7 using the polyethylene glycol/lithium acetate method. The co-transformed yeasts were grown in SD -Leu/-Trp, SD -Leu/-Trp/-His and SD -Leu/-Trp/-His/-Ade for testing protein-protein interaction through the activation of the two reporter genes *His3* and *Ade2*. The strength of the interaction was assayed by drop test using serial dilutions of mid-exponential-phase cultures. 3 µl drops of undiluted, 10- and 100-fold diluted culture were spotted on the selective agar medium and incubated at 30°C for 2 days. Details of the primers used for plasmid construction are shown in Table S2. Plasmid constructs are as shown in Figure 6.

## Supporting Information

Figure S1 A. Peptides from *Brassica oleracea* meiocytes with homology to gene At2g46980 *(AtASY3)* identified by mass spectrometry. Peptides were identified in two independent experiments using the procedure described in Sanchez-Moran *et al.*, 2005 Cytogenet and Genome Res. 109:181–189 [14]. The *Arabidopsis* TAIR database was used for peptide identification. B. (i) Diagrammatic representation of the 793 aa, 88 kDa AtASY3 protein indicating the relative position of the putative coiled-coil domain (black box). (ii) Map of the ∼3.5 kb At2g46980 locus showing the exon/intron organization of *AtASY3*. The exons are represented by numbered black boxes. The triangles indicate the T-DNA insertion sites in *Atasy3-1*, *Atasy3-2* and *Atasy3-3*. C. Expression analysis of *AtASY3* using semi-quantitative RT-PCR indicates that in wild-type (WT) expression is highest in bud tissue (B) with a low level present in open flowers (F). Expression is not detected in stem (S) or leaf (L). *AtASY3* expression is absent in the *Atasy3-1*, *Atasy3-2* and *Atasy3-3* mutants. *AtGAPD* was used as an expression control.
(PDF)

Figure S2 Nucleotide sequencing of the T-DNA insertion sites in *Atasy3-1*, *Atasy3-2* and *Atasy3-3*.
(PDF)

Figure S3 A. Fertility in *Atasy3-1* is reduced compared to wild-type Col 0. (A) wild-type, (B) *Atasy3-1*, (C) wild-type silique, (D) *Atasy3-1* silique. Bar 10 mm. *Atasy3-1* exhibited a reduction in mean silique length of 37% (n = 50) and a reduction in seed-set of 73% (n = 50). B. Representative meiotic stages of *Atasy3-2* (A–F) and *Atasy3-3* (G–L). Leptotene (A,G); pachytene (B,H); diakinesis (C,I); metaphase (D,J); dyad (E,K) tetrad (F,L). Bar, 10 µm. C. An allelism test was carried out by reciprocally crossing heterozygous *Atasy3-1* and *Atasy3-2*. Cytological analysis of *Atasy3-1/Atasy3-2* reveals asynapsis at pachytene (A) and univalents in metaphase I (B). This leads to mis-segregation at meiotic divisions resulting in the subsequent formation of unbalanced tetrads (C). Bar, 10 µm. Fertility in an *Atasy3-1* complementation line (F) was restored to the normal level observed in wild-type (D) in contrast to that of *Atasy3-1* (E). Bar, 10 mm. Cytological analysis confirmed that normal meiosis was restored in the *Atasy3-1* complementation line. Homologous chromosomes underwent normal synapsis in pachytene (G). A full complement of five bivalents was observed in metaphase I (H). These underwent normal segregation leading to the formation of balanced tetrads (I). Bar, 10 µm.
(PDF)

Figure S4 A. Chromosome spread preparations from PMCs at metaphase I were examined by light microscopy after fluorescence *in situ* hybridization (FISH) using 45S (green) and 5S (red) rDNA probes. The use of FISH enabled the identification of individual chromosomes. The overall shape of individual bivalents allowed the number and position of individual chiasmata to be determined and this was also informed by the position of the FISH signals. For full details of the chiasma scoring procedure see: Sanchez-Moran *et al.* (2002) Genetics 162: 1415–1422 [58]. Analyses of metaphase I nuclei of wild-type (A) a-b. Rod bivalents, single interstitial chiasma in the long arm Chr. 2 and Chr. 4 respectively; c-e. Ring-bivalents, 2 chiasmata Chr. 1, Chr 5 and Chr.3 respectively. *Atasy3-1* (B) a. Chr. 5 rod bivalent distal chiasma; b. Chr. 1 rod bivalent distal chiasma, c. Chr. 4 rod bivalent single short arm chiasma. Analysis indicated that mean chiasma frequency in *Atasy3-1* was significantly reduced to 3.40 in contrast to wild-type, which had an overall mean chiasma frequency of 9.84. Bar, 10 µm. B. Cytological analyses of metaphase I chromosome spreads indicated the presence of univalents in *Atasy3-1/Atspo11-1-4* (A). No chiasmata were observed in this double mutant in contrast to wild-type (B), where five bivalents were observed in all of the metaphase I cells analysed. This confirms that the chiasmata in *Atasy3-1* are DSB-dependent. Comparison of the mean chiasma frequency of *Atasy3-1/Atasy1* (C) and *Atasy1* (D) revealed no significant difference between the double mutant and the latter suggesting a close functional relationship between AtASY3 and AtASY1. Analysis of 30 metaphase I nuclei from *Atasy3-1/Atmsh4* (E) shows that the double mutant fails to form chiasmata in contrast to *Atmsh4* (F), in which a mean chiasma frequency of 1.1 (n = 30) was observed. Bar, 10 µm.
(PDF)

Figure S5 Immunolocalization of recombination proteins in *Atasy3-1* and γH2AX *in Atasy1*. Immunolocalization of AtRAD51 (red) on DAPI stained (blue) wild-type (A) and *Atasy3-1*(B) meiocytes at early prophase I. (C) and (D) show corresponding images for AtMSH4. In both cases there is a reduction in foci in the *Atasy3-1* mutant. Immunolocalization of γH2AX (red) on DAPI stained (blue) meiocytes from wild-type (E) and *Atasy1* (F). *(see main text for details)*. *Bar*, 10 µm.
(PDF)

Figure S6 Time-course analysis of AtDMC1 (red) localization in wild-type (WT), *Atasy1* and *Atasy3-1*. The study revealed that AtDMC1 foci in *Atasy3-1* are stabilized and persist at least up to 24 h post BrdU (green) pulse-labeling at S phase before gradually decreasing to very low numbers by 30 h. This observation was similar to that in WT but contrast with that of *Atasy1*, where AtDMC1 foci are destabilized soon after loading and their numbers decrease rapidly at ∼18 h. Almost all of AtDMC1 foci in *Atasy1* were lost by 24 h post BrdU pulse labeling as previously reported by Sanchez-Moran *et al.* 2007 [8].
(PDF)

Figure S7 Sequence alignment of AtASY3 and BoASY3. The proteins exhibit 77% sequence identity.
(PDF)

Figure S8 Immunolocalization of BoASY3 protein using anti-AtASY3 antibody to wild-type *Brassica oleracea* chromosome spread preparations from meiocytes at leptotene, zygotene and pachytene. The BoASY3 protein localises to meiotic chromosomes as numerous foci in leptotene and gradually polymerizes to form a continuous linear signal by pachytene. The localisation of BoASY3 is indistinguishable to that of AtASY3 in *Arabidopsis*. BoASY3 could not be detected using pre-immune anti-AtASY1 antiserum. Bar, 10 µm.
(PDF)

Figure S9 A. BoASY3 sequence showing protein coverage following co-immunoprecipitation with BoASY1 and MS analysis (highlighted yellow). B. Mass spectrometry conditions.
(PDF)

Table S1 Chiasma counts for *Atasy3-1* and wild-type. A. *Atasy3-1* mean chiasma frequency 3.3 (n = 50). Proportion of distal chiasma = 74.8%. B. *Arabidopsis* (Col-0) wild-type mean chiasma frequency 9.76 (n = 50). Proportion of distal chiasma = 73.8%. The proportion of distal chiasmata is not significantly different in the mutant.
(PDF)

Table S2 Primer sequences used during the study. Primers 1–8 were used for confirming T-DNA insertions sites; primers 9–12 were used for the analysis of *AtASY3* expression; Primers 13 and 14 were used for complementation studies; primers 15 and 16 were used for the production of recombinant AtASY3 for antibody production. Primers 17–22 were for yeast two-hybrid studies. The regions amplified are indicated; F/R denotes forward and reverse.
(PDF)
