# Supplementary material for: Inter-Homolog Crossing-Over and Synapsis in Arabidopsis Meiosis Are Dependent on the Chromosome Axis Protein AtASY3
Source: PLoS Genet. 2012 Feb 2;8(2):e1002507. doi: 10.1371/journal.pgen.1002507 (PMC3271061; doi:10.1371/journal.pgen.1002507)

Ferdous_FigS1

A.

Peptide	Sequence	Position in At2g46980
1	KISIGVMADSQPKRN	19-33
2	SATVTELQANK	52-62
3	EKSDLAAK	64-71
4	MEEPPSAVLQQKVASQR	163-179

B. (i)

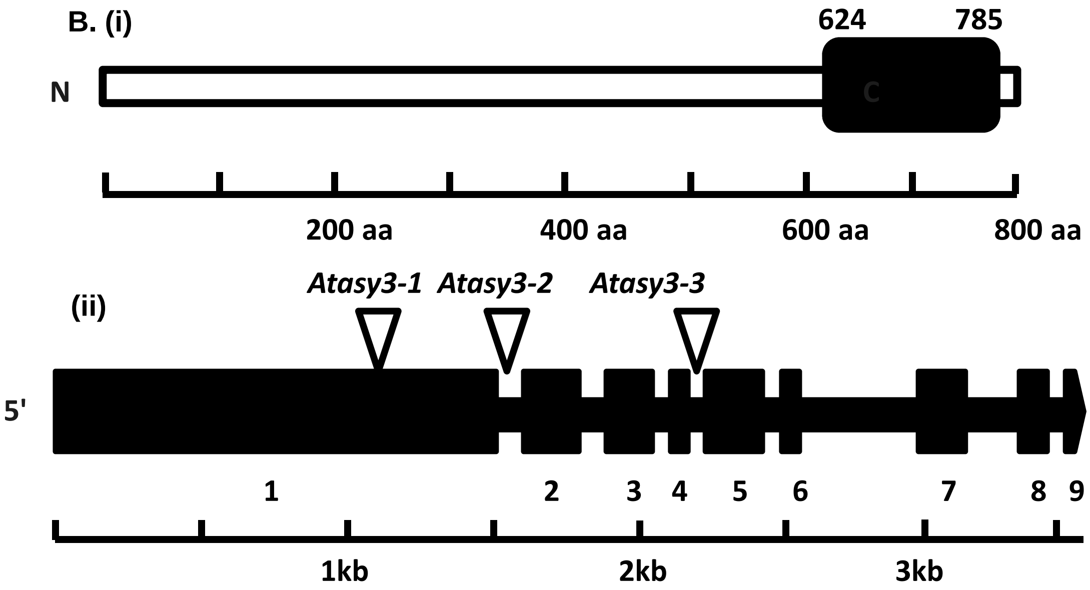

C.

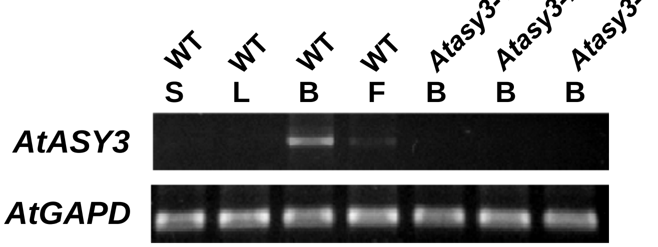

Supplement: Figure S1 — A. Peptides from Brassica oleracea meiocytes with homology to gene At2g46980 (AtASY3) identified by mass spectrometry. Peptides were identified in two independent experiments using the procedure described in Sanchez-Moran et al., 2005 Cytogenet and Genome Res. 109:181–189 [14]. The Arabidopsis TAIR database was used for peptide identification. B. (i) Diagrammatic representation of the 793 aa, 88 kDa AtASY3 protein indicating the relative position of the putative coiled-coil domain (black box). (ii) Map of the ∼3.5 kb At2g46980 locus showing the exon/intron organization of AtASY3. The exons are represented by numbered black boxes. The triangles indicate the T-DNA insertion sites in Atasy3-1, Atasy3-2 and Atasy3-3. C. Expression analysis of AtASY3 using semi-quantitative RT-PCR indicates that in wild-type (WT) expression is highest in bud tissue (B) with a low level present in open flowers (F). Expression is not detected in stem (S) or leaf (L). AtASY3 expression is absent in the Atasy3-1, Atasy3-2 and Atasy3-3 mutants. AtGAPD was used as an expression control. (PDF) [file pgen.1002507.s001.pdf]
