# Supplementary material for: Inter-Homolog Crossing-Over and Synapsis in Arabidopsis Meiosis Are Dependent on the Chromosome Axis Protein AtASY3
Source: PLoS Genet. 2012 Feb 2;8(2):e1002507. doi: 10.1371/journal.pgen.1002507 (PMC3271061; doi:10.1371/journal.pgen.1002507)
Supplement: Figure S2 — Nucleotide sequencing of the T-DNA insertion sites in Atasy3-1, Atasy3-2 and Atasy3-3. (PDF) [file pgen.1002507.s002.pdf]

Ferdous_FigS2

A **SALK_143676 *(Atasy3-1)***

CTGGTGCCAACTTAGGTCGCAAGTGCACAGAACAAGTAAATAGTGTATTTTCTTTTGAGGAAGGTTTGC
GCGGAAAAATTGGCACTGCTGTGAATTCCAGTGTTATGCCAAAGAAACAAAGGGGTAGAAGAAAAAACA
CTGTTGTAAAATGCCGTAAGGCTCATTCTCGAAAAAAGGATGAAGCTGATTGGAGTCGGAAGGAGGCGA
GCAAGAGCAATACTCCACCACGTTCTGAAAGCACAGAAACTGGCAAAAGATCTTCATCTTCAGACAAAA
AGGGAAGTTCCCATGACCTTCATCCACAGAGCAAAGCCCGGAAACAGAAACCAGATATTGTGCTAATAT
CACAACTTGACGCTTAGACAACTTAATAACACATTGCGGACGTTTTTAATGTACTGGGGTGGTTTTTCT
TTTCACCAGTGAGACGGGCAACAGCTGATTGCCCTTCACCGCCTGGCCCTGAGAGAGTTGCAGCAAGCG
GTCCACGCTGGTTTGCCCCAGCAGGCGAAAATCCTGTTTGATGGTGGTTCCGAAATCGGCAAAAT

Primers: LBb1.3 + ASY3-1-R1

ASY3 genomic DNA
Sequence from TDNA – insertion position = between DNA residues 188 and 1189 in exon 1.

B **SAIL_423_H01**

GCAAGAGCAATACTCCACCACGTTCTGAAAGCACAGAAACTGGCAAAAGATCTTCATCTTCAGACAAAA
AGGGAAGTTCCCATGACCTTCATCCACAGAGCAAAGCCCGGAAACAGAAACCAGATATTAGCACAAGGG
AAGGAGATTTTCATCCATCGCCAGAAGCTGAGGCAGCAGCTCTGCCAGAGATGTCCCAGGGATTATCTA
AAAATGGCGATAAACATGAACGGCCGAGTAATATTTTCAGGGAAAAGTCTGTTGAGCCAGAAAACGAAT
TCCAGAGTCCAACCTTTGGATATAAAGCACCAATCTCAAGTCCTTCCCCATGTTGTTCTCCAGAAGCAT
CTCCTTTGCAACCTAGGAATATTAGTCCCACATTAGATGAGACGGAAACACCAATATTTAGCTTCGGTA
CTAAGAAAACCTCTCAAGGGACAACAGGCCAAGCGTCAGATACAGAAAAGAGATTGCCTGTAACCTTCA
TAAACTGAAGCGGCTTTATCTATACTTAAGTTGCATATTGTGGTGTAAACAAATTGACGCTTAGACAAC
TTAATAACACATTGTGGACGTTTTTAATGTACTGAATTAACGCCGAATTGAATTCGATTTGGTGTATCG
AGATTGGTTATGAA

Primers: LB3 + ASY3-2-R1
ASY3 genomic DNA
Sequence from TDNA – insertion position = between DNA residues 1583 and 1584 in intron 1.

C **SALK_050971**

GATTATTTTGCCGATTTCGGAACCACCATCAAACAGGATTTTCGCCTGCTGGGGCAAACCAGCGTGGAC
CGCTTGCTGCAACTCTCTCAGGGCCAGGCGGTGAAGGGCAATCAGCTGTTGCCCGTCTCACTGGTGAAA
AGAAAAACCACCCCAGTACATTAAAAACGTCCGCAATGTGTTATTAAGTTGTCTAAGCGTCAATTTGTT
TACACCACAATATATCCTGTCTTATTAGTTATACCTACAAGAGACCTTCCCAAGCCTTCATCTTCATCC
TCATCCATCTCTGAACAATGCTGGAAGGAATCAGTTTTATCGATCCCTGCTCAGTTTCCAGGACAAAAC
AAATGAGGGTGCAGTATATAAACCCACAACAGTGACATACAAATGAGCATTATACCTTTTGACAAAGGG
GAGGGAGGACTTAACACGACACGTCCAATGCCCTTAAGGTTAGAGTCCCGTTTAACTGAGCTAGGGCCT
AACATAGATCTCTCATTAGTCCAATTGGCAGTTTCTCTTTCTTCGGGGCCTGAATCAGAAGATCAATAG
AATAAGCAAAACATGGATTCTGTTAAGAAGAAAATAAGATCCTCCAGTTTTTATATGGTCTAGGATAAG
AATTAAGAAGCATACTGATGTTATGGCCTAACACTGGTGAATCTTCTCTTGAACCATCTGAGTCTCGC

Primers: LBb1.3 + ASY3-3-R1
ASY3 genomic DNA
Sequence from TDNA – insertion position = between DNA residues 2186 and 2187 in intron 4.
