# Supplementary figures and images for: Inter-Homolog Crossing-Over and Synapsis in Arabidopsis Meiosis Are Dependent on the Chromosome Axis Protein AtASY3
Source: PLoS Genet. 2012 Feb 2;8(2):e1002507. doi: 10.1371/journal.pgen.1002507 (PMC3271061; doi:10.1371/journal.pgen.1002507)

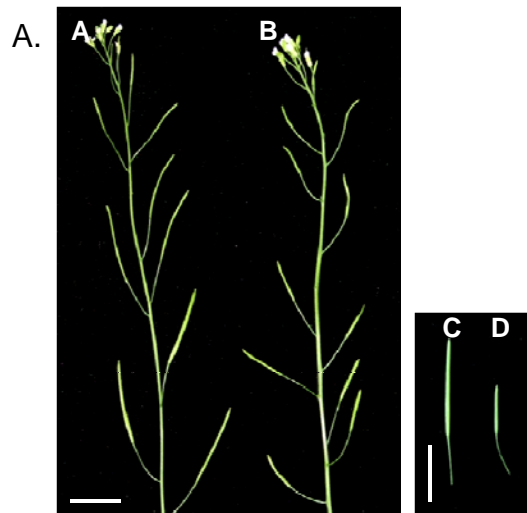

B.

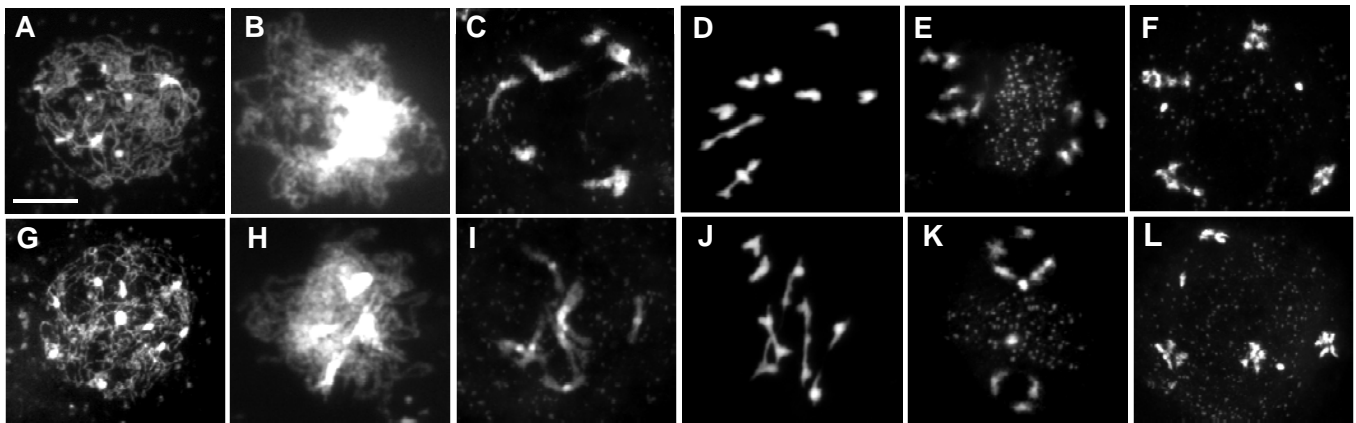

C.

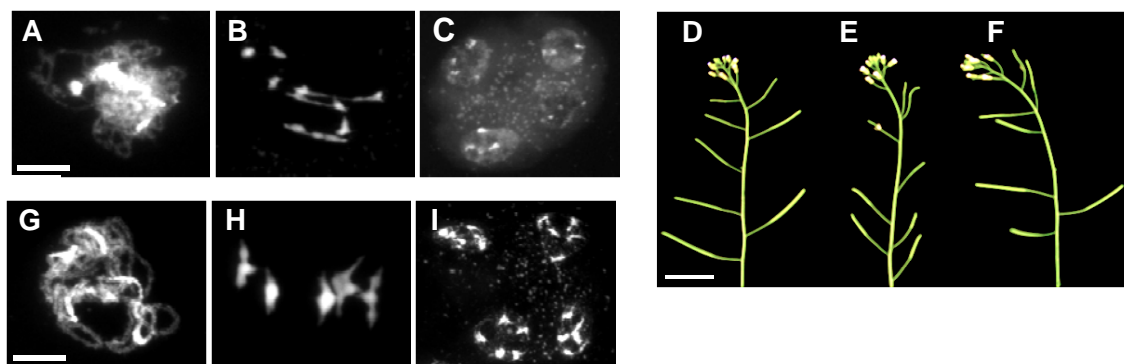

Supplement: Figure S3 — A. Fertility in Atasy3-1 is reduced compared to wild-type Col 0. (A) wild-type, (B) Atasy3-1, (C) wild-type silique, (D) Atasy3-1 silique. Bar 10 mm. Atasy3-1 exhibited a reduction in mean silique length of 37% (n = 50) and a reduction in seed-set of 73% (n = 50). B. Representative meiotic stages of Atasy3-2 (A–F) and Atasy3-3 (G–L). Leptotene (A,G); pachytene (B,H); diakinesis (C,I); metaphase (D,J); dyad (E,K) tetrad (F,L). Bar, 10 µm. C. An allelism test was carried out by reciprocally crossing heterozygous Atasy3-1 and Atasy3-2. Cytological analysis of Atasy3-1/Atasy3-2 reveals asynapsis at pachytene (A) and univalents in metaphase I (B). This leads to mis-segregation at meiotic divisions resulting in the subsequent formation of unbalanced tetrads (C). Bar, 10 µm. Fertility in an Atasy3-1 complementation line (F) was restored to the normal level observed in wild-type (D) in contrast to that of Atasy3-1 (E). Bar, 10 mm. Cytological analysis confirmed that normal meiosis was restored in the Atasy3-1 complementation line. Homologous chromosomes underwent normal synapsis in pachytene (G). A full complement of five bivalents was observed in metaphase I (H). These underwent normal segregation leading to the formation of balanced tetrads (I). Bar, 10 µm. (PDF) [file pgen.1002507.s003.pdf]

A.

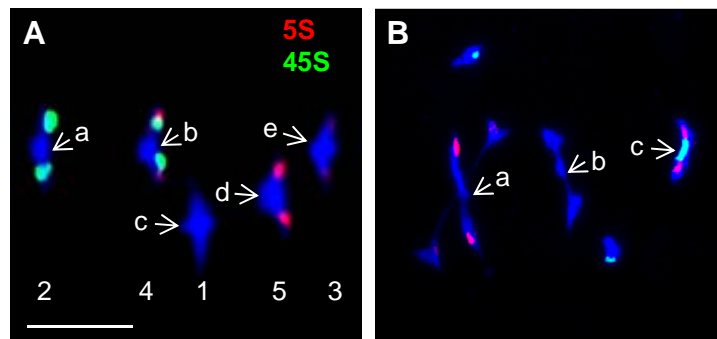

B.

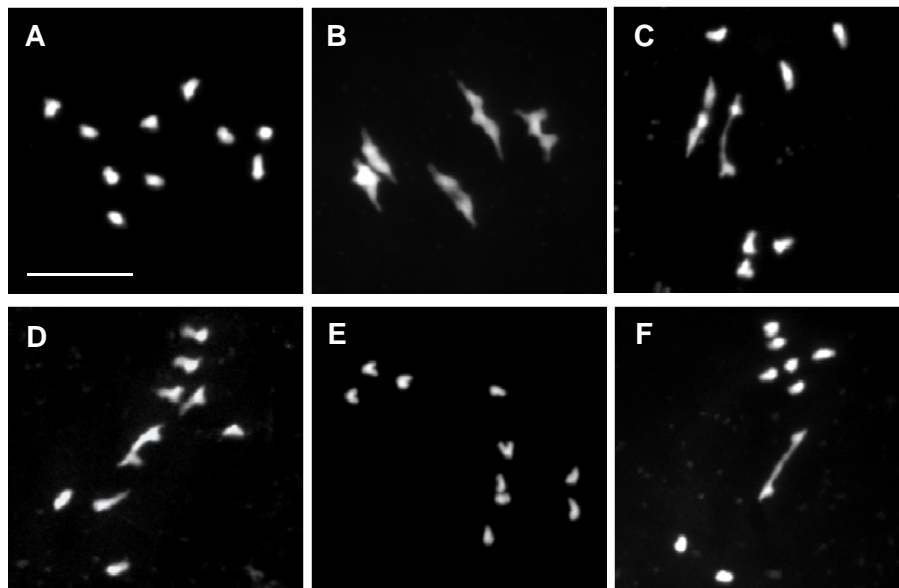

Supplement: Figure S4 — A. Chromosome spread preparations from PMCs at metaphase I were examined by light microscopy after fluorescence in situ hybridization (FISH) using 45S (green) and 5S (red) rDNA probes. The use of FISH enabled the identification of individual chromosomes. The overall shape of individual bivalents allowed the number and position of individual chiasmata to be determined and this was also informed by the position of the FISH signals. For full details of the chiasma scoring procedure see: Sanchez-Moran et al. (2002) Genetics 162: 1415–1422 [58]. Analyses of metaphase I nuclei of wild-type (A) a-b. Rod bivalents, single interstitial chiasma in the long arm Chr. 2 and Chr. 4 respectively; c-e. Ring-bivalents, 2 chiasmata Chr. 1, Chr 5 and Chr.3 respectively. Atasy3-1 (B) a. Chr. 5 rod bivalent distal chiasma; b. Chr. 1 rod bivalent distal chiasma, c. Chr. 4 rod bivalent single short arm chiasma. Analysis indicated that mean chiasma frequency in Atasy3-1 was significantly reduced to 3.40 in contrast to wild-type, which had an overall mean chiasma frequency of 9.84. Bar, 10 µm. B. Cytological analyses of metaphase I chromosome spreads indicated the presence of univalents in Atasy3-1/Atspo11-1-4 (A). No chiasmata were observed in this double mutant in contrast to wild-type (B), where five bivalents were observed in all of the metaphase I cells analysed. This confirms that the chiasmata in Atasy3-1 are DSB-dependent. Comparison of the mean chiasma frequency of Atasy3-1/Atasy1 (C) and Atasy1 (D) revealed no significant difference between the double mutant and the latter suggesting a close functional relationship between AtASY3 and AtASY1. Analysis of 30 metaphase I nuclei from Atasy3-1/Atmsh4 (E) shows that the double mutant fails to form chiasmata in contrast to Atmsh4 (F), in which a mean chiasma frequency of 1.1 (n = 30) was observed. Bar, 10 µm. (PDF) [file pgen.1002507.s004.pdf]

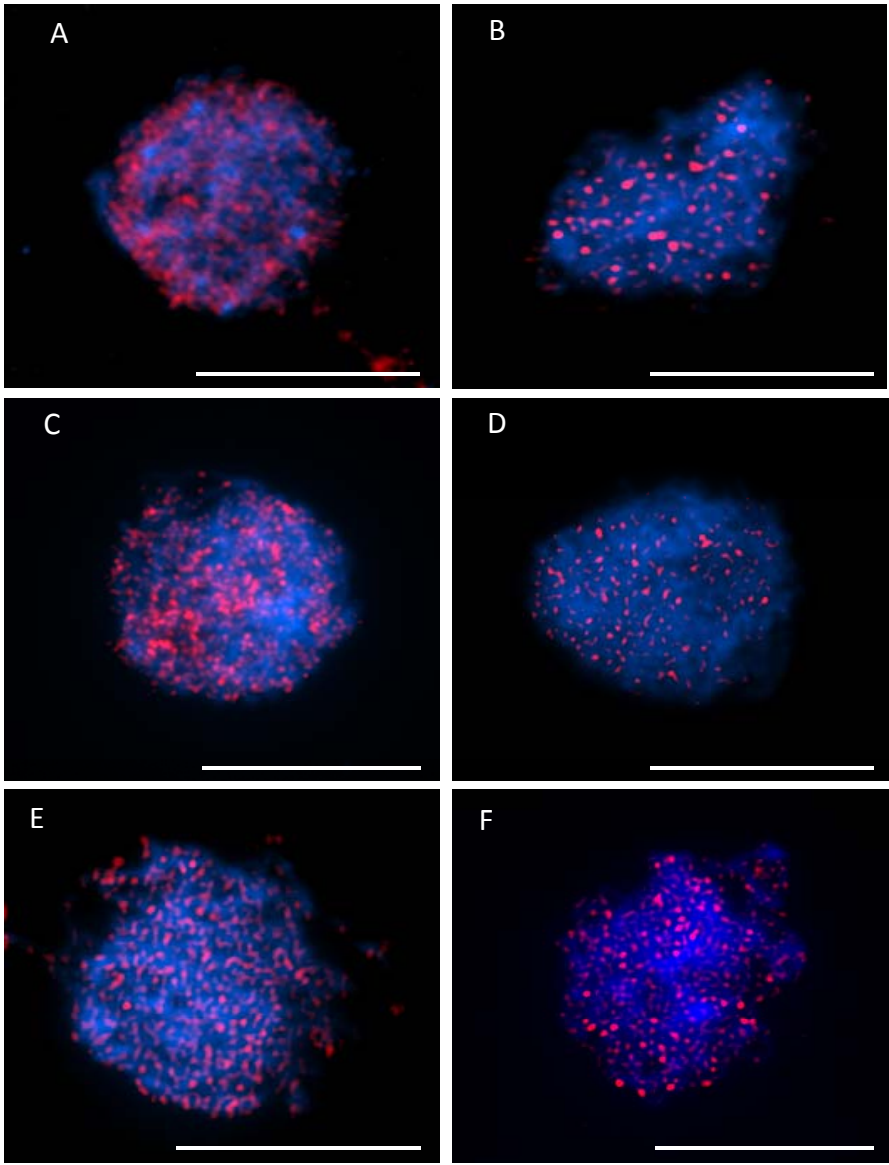

Supplement: Figure S5 — Immunolocalization of recombination proteins in Atasy3-1 and γH2AX in Atasy1. Immunolocalization of AtRAD51 (red) on DAPI stained (blue) wild-type (A) and Atasy3-1(B) meiocytes at early prophase I. (C) and (D) show corresponding images for AtMSH4. In both cases there is a reduction in foci in the Atasy3-1 mutant. Immunolocalization of γH2AX (red) on DAPI stained (blue) meiocytes from wild-type (E) and Atasy1 (F). (see main text for details). Bar, 10 µm. (PDF) [file pgen.1002507.s005.pdf]

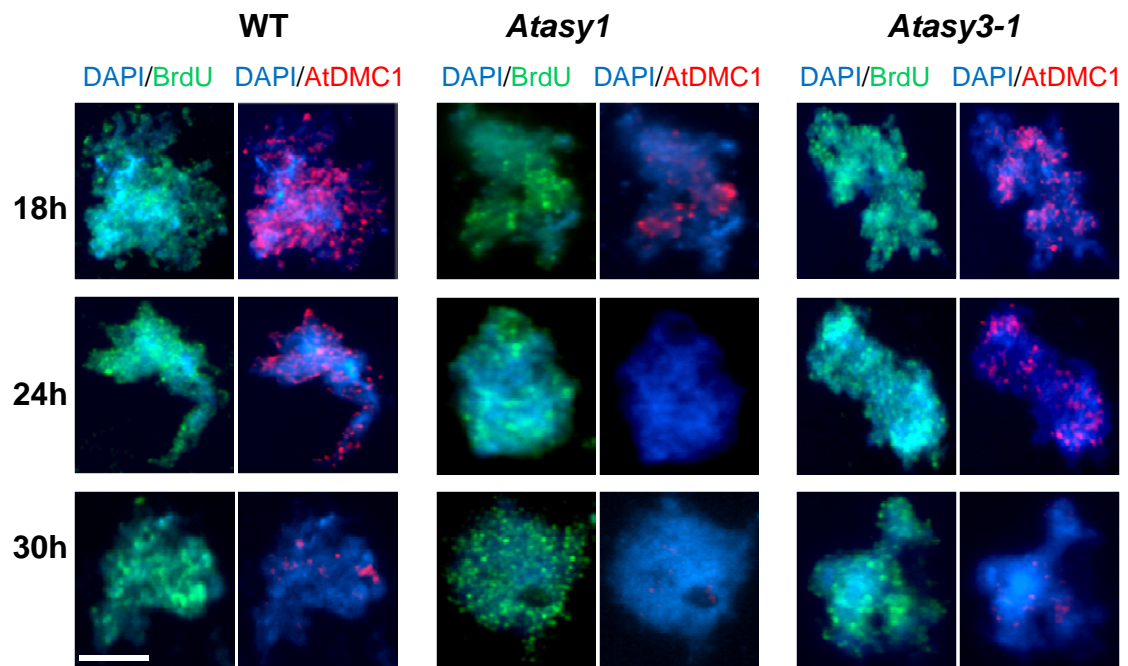

Supplement: Figure S6 — Time-course analysis of AtDMC1 (red) localization in wild-type (WT), Atasy1 and Atasy3-1. The study revealed that AtDMC1 foci in Atasy3-1 are stabilized and persist at least up to 24 h post BrdU (green) pulse-labeling at S phase before gradually decreasing to very low numbers by 30 h. This observation was similar to that in WT but contrast with that of Atasy1, where AtDMC1 foci are destabilized soon after loading and their numbers decrease rapidly at ∼18 h. Almost all of AtDMC1 foci in Atasy1 were lost by 24 h post BrdU pulse labeling as previously reported by Sanchez-Moran et al. 2007 [8]. (PDF) [file pgen.1002507.s006.pdf]

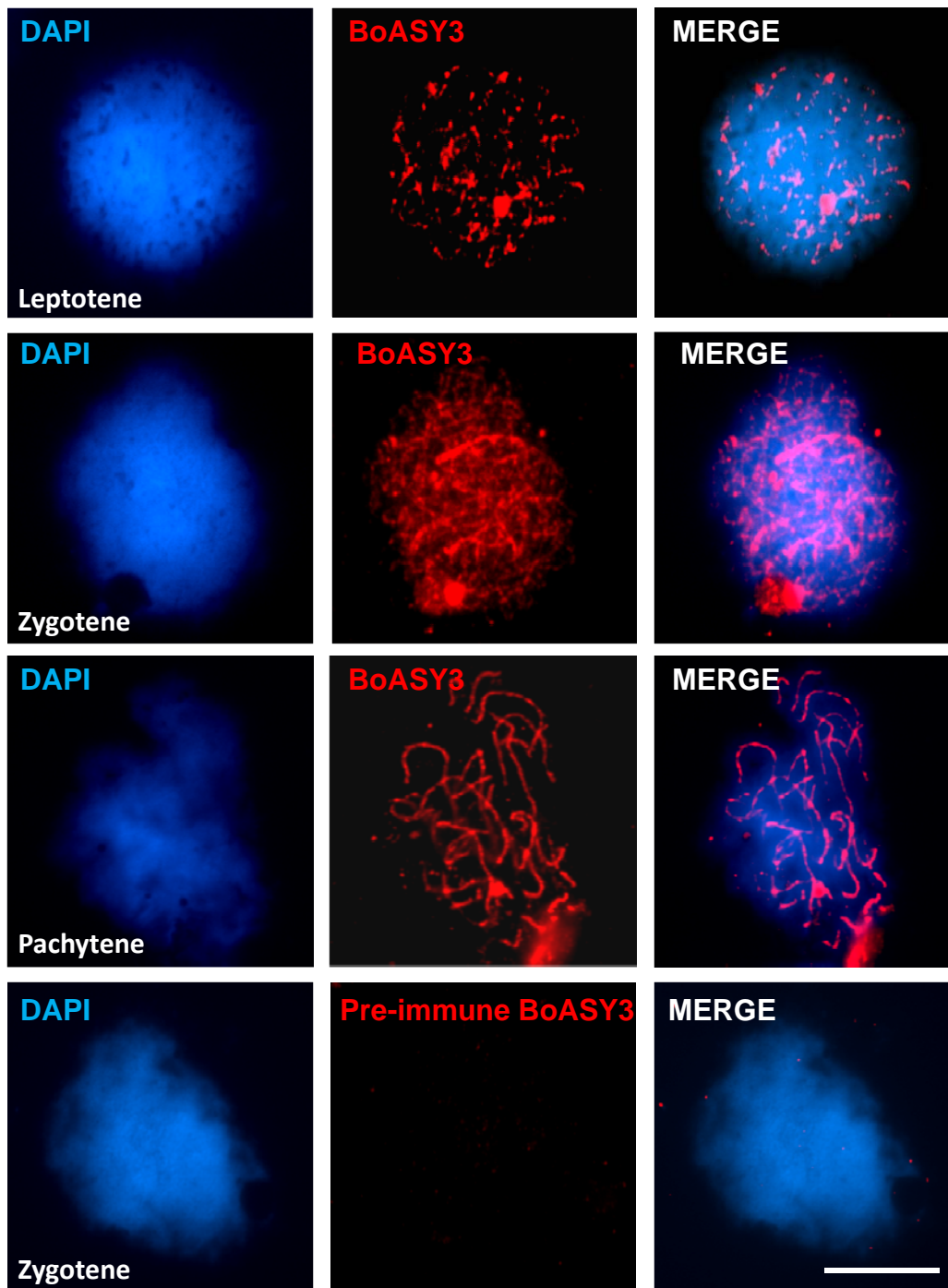

Supplement: Figure S8 — Immunolocalization of BoASY3 protein using anti-AtASY3 antibody to wild-type Brassica oleracea chromosome spread preparations from meiocytes at leptotene, zygotene and pachytene. The BoASY3 protein localises to meiotic chromosomes as numerous foci in leptotene and gradually polymerizes to form a continuous linear signal by pachytene. The localisation of BoASY3 is indistinguishable to that of AtASY3 in Arabidopsis. BoASY3 could not be detected using pre-immune anti-AtASY1 antiserum. Bar, 10 µm. (PDF) [file pgen.1002507.s008.pdf]
