# Supplementary material for: Inter-Homolog Crossing-Over and Synapsis in Arabidopsis Meiosis Are Dependent on the Chromosome Axis Protein AtASY3
Source: PLoS Genet. 2012 Feb 2;8(2):e1002507. doi: 10.1371/journal.pgen.1002507 (PMC3271061; doi:10.1371/journal.pgen.1002507)
Supplement: Figure S7 — Sequence alignment of AtASY3 and BoASY3. The proteins exhibit 77% sequence identity. (PDF) [file pgen.1002507.s007.pdf]

Ferdous_FigS7

```
AtASY3          MSDYRSFGSNYHPSSQSRKISIGVMADSQPKRNLVPDKDDGDVIARVEKLKSATVTELQA 60
BoASY3          MSEYRSFGSNFHPSSQPRKMSIGVMADSQPKR----HPDGAAAIGRAEKLKSAAATDLQL 56
                **:*******:*****.**:************     . *.. .*.*.******:.*:**

AtASY3          NKKEK-SDLAAKQRNSAQVTGHVTSPWRSPRSSHRKLGTLESVLCKQTSSLSGSKGLNKG 119
BoASY3          NKKVTGDDVAAKQRSSAKGTDHVTSPWRSPRSSYRKLGTLENVLCKQTSSLSGSKGLNKG 116
                ***  . .*:*****.**: *.************:*******.******************

AtASY3          LNGAHQTPARESFQNCPISSPQHSLGELNGGRNDRVMDRSPERMEEPPSAVLQQKVASQR 179
BoASY3          PNGAHQAPARDSFQDIPVSSPRHSDDEPISGRKGNEMDKSPERMQEPPSAVLQQKVASQR 176
                 *****:***:***: *:***:** .*  .**:.. **:*****:***************

AtASY3          EKMDKPGKETNGTTDVLRSKLWEILGKASPANNEDVNSETPEVEKTNFKLSQDKGSNDDP 239
BoASY3          EEKRGPETAKDGSTDVLRSKLWEILGKASPEYNEDVNSETPEVVKTNSKLNQDKTSNDDP 236
                *:   * . .:*:*****************  *********** *** **.*** *****

AtASY3          LIKPRHNSDSIETDSESPENATRRPVTRSLLQRRVGAKGVQKKTKAGANLGRKCTEQVNS 299
BoASY3          LTKPRHHSDTIETDSESPEVATRRPVTRSLLQRRVGARGIQKRTKTGANLGGKSTEEVNN 296
                * ****:**:********* *****************:*:**:**:***** *.**:**.

AtASY3          VFSFEEGLRGKIGTAVNSSVMPKKQRGRRKNTVVKCRKAHSRKKDEADWSRKEASKSNTP 359
BoASY3          VFTFEEGLRGRNG----TTVMPKKQRGRKKNTAVKCRKVQSREKEEADGILKETSKSKTP 352
                **:*******: *    ::*********:***.*****.:**:*:***    **:***:**

AtASY3          PRSESTETGKRSSSSDKKGSSHDLHPQSKARKQKPDISTREGDFHPSPEAEAAALPEMSQ 419
BoASY3          ARSESTRTGKRSSLSDKKGSSLEFNQHTKAQKQKQDVSTREEDFQPSPEAETAATPEMFR 412
                .*****.****** ******* ::: ::**:*** *:**** **:******:** *** :

AtASY3          GLSKNGDKHERPSNIFREKSVEPENEFQPSPTFGYKAPISSPSPCCSPEASPLQPRNISPT 479
BoASY3          GLFKNGDEQKGPCEVLREKSVEPENDFQPSPTFGYKAPISSPSPCFSPEASPLHPRNISPA 472
                ** ****::: *.:::*********:****************** *******:******:

AtASY3          LDETETPIFSFGTKKTSQGTTGQASDTEKRLPDFLEKKRDYSFRRESSPEPNEDLVLSDP 539
BoASY3          FDETETAIFSFGTKRTPQETKGQVSD--KRLPDFLEKKGDYSFGRESSAEPDEDLVLSDP 530
                :*****.*******:*.* *.**.**  ********** **** ****.**:********

AtASY3          SSDERDSDGSREDSPVLGHNISPEERETANWTNERSMLGPSSVKRNSNLKGIGRVVLSPP 599
BoASY3          SSDEKDSDGSIEDS----HYNNPQVRETANGSNKKSKQGFGSAKRNSNLKGNGRVTSS-- 584
                ****:***** ***    *  .*: ***** :*::*  * .*.******** ***. *

AtASY3          SPLSKGIDKTDSFQHCSEMDEDEDEGLGRAVALFAMALQNFERKLKSAAEKKSSEIIASV 659
BoASY3          --LSEGMHKTDSFQRFSEVDEDE--GMGRAVALFAVALQNFEKKLKSAAKKKSSEIIASV 640
                  **:*:.******: **:****  *:********:******:******:**********

AtASY3          SEEIHLELENIKSHIITEAGKTSNLAKTKRKHAETRLQEQEEKMRMIHEKFKDDVSHHLE 719
BoASY3          SEEIHLELENVKSHIITEAEKTSNVAKTKRKHAETRLQEQQEKMRMIHEKFKDDVGNHLE 700
                **********:******** ****:***************:**************.:***

AtASY3          DFKSTIEELEANQSELKGSIKKQRTSHQKLIAHFEGGIETKLDDATKRIDSVNKSARGKM 779
BoASY3          DFKSTIEGLEANHSELKGSIKKQRTSHQKLIAHFEGGIETKLDNATKRINSVNESARGKM 760
                ******* ****:******************************:*****:***:******

AtASY3          LQLKMIVAECLRDD-- 793
BoASY3          LQLKMIVAECLKDDVC 776
                ***********:**
```
