# Supplementary material for: Inter-Homolog Crossing-Over and Synapsis in Arabidopsis Meiosis Are Dependent on the Chromosome Axis Protein AtASY3
Source: PLoS Genet. 2012 Feb 2;8(2):e1002507. doi: 10.1371/journal.pgen.1002507 (PMC3271061; doi:10.1371/journal.pgen.1002507)
Supplement: Figure S9 — A. BoASY3 sequence showing protein coverage following co-immunoprecipitation with BoASY1 and MS analysis (highlighted yellow). B. Mass spectrometry conditions. (PDF) [file pgen.1002507.s009.pdf]

Ferdous_FigS9

A.

```
MSEYRSFGSNFHPSSQPRKMSIGVMADSQPKRHPDGAAAIGRAEKLKSAAATDLQLNKKVTGDDVAAK
QRSSAKGTDHVTSPWRSPRSSYRKLGTLENVLCKQTSSLSGSKGLNKGPNGAHQAPARDSFQDIPVSS
PRHSDDEPISGRKGNEMDKSPERMQEPPSAVLQQKVASQREEKRGPETAKDGSTDVLRSKLWEILGKA
SPEYNEDVNSETPEVVKTNSKLNQDKTSNDDPLTKPRHHSDTIETDSESPEVATRRPVTRSLLQRRVG
ARGIQKRTKTGANLGGKSTEEVNNVFTFEEGLRGRNGTTVMPKKQRGRKKNTAVKCRKVQSREKEEAD
GILKETSKSKTPARSESTRTGKRSSLSDKKGSSLEFNQHTKAQKQKQDVSTREEDFQPSPEAETAATP
EMFRGLFKNGDEQKGPCEVLREKSVEPENDFQSPTFGYKAPISSPSPCFSPEASPLHPRNISPAFDET
ETAIFSFGTKRTPQETKGQVSDKRLPDFLEKKGDYS[illegible]GRESSAEPDEDLVLSDPSSDEKDSDGSIEDS
HYNNPQVRETANGSNKKSKQGFGSAKRNSNLKGNGRVTSSLSEGMHKTDSFQRFSEVDEDEGMGRAVA
LFAVALQNFEKKLKSAAKKKSSEIIASVSEEIHLELENVKSHIITEAEKTSNVAKTKRKHAETRLQEQ
QEKMRMIHEKFKDDVGNHLEDFKSTIEGLEANHSELKGSIKKQRTSHQKLIAHFEGGIETKLDNATKR
INSVNESARGKMLQLKMIVAECLKDDVC
```

B.

Proteins were eluted from the antibody-coupled beads with 2 bead volumes of 0.1M glycine and the pH was adjusted to 8 by addition of a few µl of 1.5M Tris-HCl pH 9.2. Proteins were reduced with dithiothreitol and alkylated with Methyl methanethiosulfonate (MMTS) and digested with 800ng trypsin over night at 37°C. Protein digests were acidified with trifluoroacteic acid (TFA).

Peptide samples were applied to an Ultimate 3000 dual gradient nano-LC system (Dionex) which was coupled online to the mass spectrometer. The samples were applied to a reversed-phase trap column for desalting using 0.1% TFA at a flow rate of 25 µl/min. Separation of peptides was performed on an analytical separation column (PepMap C18, 75 µm ID × 250 mm length, particle size 3 µm, pore size 100 Å), which was equilibrated in buffer A (0.1% formic acid (FA), 5% acetonitrile (ACN)).

Peptides were eluted at a flow rate of 275 nl/min by applying a linear gradient from 0 to 100% B (0.08% FA, 30% ACN) in 85 min, followed by a gradient to 85% B and 15 % C (0.08% FA, 80% ACN, 10% Trifluoroethanol) in 5 min and another gradient to 10% B and 90% C in 5 min.

The peptides eluting from the nano-RP-HPLC were analyzed on an LTQ-Orbitrap Velos mass spectrometer (Thermo Fisher Scientific), equipped with a nano-electrospray ion source (Proxeon). The mass spectrometer was operated in data-dependent mode. 1 Full scan (m/z 350 - 2000) was acquired in the Orbitrap (Resolution 60,000, target value 1,000,000, maximal fill time of 200 ms) followed by MS/MS scans of the 20 most abundant ions in the LTQ. The MS/MS spectra were acquired with multistage activation enabled for neutral loss of phosphoric acid (32.66, 48.99 and 97.97). The chosen ions were excluded from further selection for 180 s. The lock mass option was enabled.

For peptide identification spectra were searched against a combined protein database containing NCBI sequences from *Brassica oleracea*, *B. napus* and *B. rapa* (Feb 2010, 5679 sequences), into which the BoASY1 and BoASY3 full-length sequences and common contaminants had been manually inserted, using Mascot Daemon 2.2.2 (Matrix Science). The following search parameters were used: Alkylation of cysteine by MMTS was set as a fixed modification, oxidation on methionine and phosphorylation on serine, threonine and tyrosine were set as variable modifications. Monoisotopic masses were searched within unrestricted protein masses for tryptic peptides. The peptide mass tolerance was set to 5 ppm and the fragment mass tolerance to 0.5 Da. The maximal number of missed cleavages was set to 2. Further analysis of the identified proteins was performed using Scaffold (version 3.0, Proteome Software Inc.).
