# Supplementary material for: Inter-Homolog Crossing-Over and Synapsis in Arabidopsis Meiosis Are Dependent on the Chromosome Axis Protein AtASY3
Source: PLoS Genet. 2012 Feb 2;8(2):e1002507. doi: 10.1371/journal.pgen.1002507 (PMC3271061; doi:10.1371/journal.pgen.1002507)
Supplement: Table S1 — Chiasma counts for Atasy3-1 and wild-type. A. Atasy3-1 mean chiasma frequency 3.3 (n = 50). Proportion of distal chiasma = 74.8%. B. Arabidopsis (Col-0) wild-type mean chiasma frequency 9.76 (n = 50). Proportion of distal chiasma = 73.8%. The proportion of distal chiasmata is not significantly different in the mutant. (PDF) [file pgen.1002507.s010.pdf]

Ferdous_Table S1

A.

Chiasma class	Chr. 1	Chr. 2	Chr. 3	Chr. 4	Chr. 5	Total
**Proximal**	2	0	0	2	1	5
**Interstitial**	10	6	26	9	5	36
**Distal**	13	18	6	32	33	122
**Total per chromosome**	25	24	32	43	39	163

B.

Chiasma class	Chr. 1	Chr. 2	Chr. 3	Chr. 4	Chr. 5	Total
**Proximal**	0	0	0	4	0	4
**Interstitial**	37	17	23	14	33	124
**Distal**	71	73	80	72	64	360
**Total per chromosome**	108	90	103	90	97	488
