# Supplementary material for: Inter-Homolog Crossing-Over and Synapsis in Arabidopsis Meiosis Are Dependent on the Chromosome Axis Protein AtASY3
Source: PLoS Genet. 2012 Feb 2;8(2):e1002507. doi: 10.1371/journal.pgen.1002507 (PMC3271061; doi:10.1371/journal.pgen.1002507)
Supplement: Table S2 — Primer sequences used during the study. Primers 1–8 were used for confirming T-DNA insertions sites; primers 9–12 were used for the analysis of AtASY3 expression; Primers 13 and 14 were used for complementation studies; primers 15 and 16 were used for the production of recombinant AtASY3 for antibody production. Primers 17–22 were for yeast two-hybrid studies. The regions amplified are indicated; F/R denotes forward and reverse. (PDF) [file pgen.1002507.s011.pdf]

Ferdous-Table S2

Primer	Sequence (5'-3')
1. LB3	TTCATAACCAATCTCGATACAC
2. LBb1.3	ATTTTGCCGATTTCGGAAC
3. ASY3-1-F1	AGGAGATGCTTCTGGAGAAC
4. ASY3-1-R1	CTGGTGCCAACTTAGGTCGC
5. ASY3-2-F1	CACGACACGTCCAATGCCC
6. ASY3-2-R1	CGAGCAAGAGCAATACTCCAC
7. ASY3-3-F1	CCAGACTCTCATGTTCCACAAC
8. ASY3-3-R1	GCGAGACTCAGATGGTTCAAG
9. ASY3-EX-F1	GAAGAGCCATAGCGAACAATGC
10. ASY3-EX-R1	CATCCACAGAGCAAAGCCCGG
11. GAPD-N	CTTGAAGGGTGGTGCCAAGAAGG
12. GAPD–C	CTGACTTCGTTGGCGACAACAGG
13. ASY3-CM-F1	CCCTCGAGAACCGACACACCATTTTGAG
14. ASY3-CM-R1	GCACTAGTATATATCAAGATATCAATAACC
15. ASY3-AB-F1	CCAATGATCAGCCCCGAAGAAAGAG
16. ASY3-AB-R1	CGCTCGAGATCATCCCTCAAACATTCTGCGAC
17. AtASY3 $^{1-793}$	F. CAG ATT ACG CTC ATA TGA GCG ACT ATA GAA GCT TC<br>R. CAC CCG GGT GGA ATT TCA ATC ATC CCT CAA ACA TTC TG
18. AtASY3 $^{1-793}$	F. AGG AGG ACC TGC AT A TGA GCG ACT ATA GAA GCT TC<br>R. GGA TCC CCG GGA ATT TCA ATC ATC CCT CAA ACA TTC TG
19. AtASY3 $^{623-793}$	F. CAG ATT ACG CTC ATG AAG GCT TGG GAA GGG CTG TT<br>R. CAC CCG GGT GGA ATT TCA ATC ATC CCT CAA ACA TTC TG
20. AtASY3 $^{1-510}$	F. CAG ATT ACG CTC ATA TGA GCG ACT ATA GAA GCT<br>R. CAC CCG GGT GGA ATT GCA ATC TCT TTT CTG TAT
21. AtASY3 $^{1-280}$	F. CAG ATT ACG CTC ATA TGA GCG ACT ATA GAA GCT TC<br>R. CAC CCG GGT GGA ATT CAG CGA CCT AAG TTG GCA CCA GC
22. AtASY1 $^{1-596}$	F. AGG AGG ACC TGC ATA TGG TGA TGG CTC AGA AGC TGA A<br>R. GGA TCC CCG GGA ATT CAA TTA GCT TGA GAT TTC TGA CGC TT
22. AtASY1 $^{1-596}$	F. CAG ATT ACG CTC ATA TGG TGA TGG CTC AGA AGC TGA A<br>R. CAC CCG GGT GGA ATT CAA TTA GCT TGA GAT TTC TGA CGC TT
